# Deciphering the Genome Evolution and Cryptic Biosynthetic Potential in Rare *Lentzea* spp.

**DOI:** 10.3390/antibiotics15100967

**Published:** 2026-09-30

**Authors:** Linqi Hua, Xingzhi Jiao, Wei Huang, Xing Fan, Wei Sun, Jie Xu, Lin Zhou, Wenjing Niu, Xiaoxia Luo, Chuanxing Wan, Linquan Bai, Qianjin Kang

**Affiliations:** 1 College of Life Science and Technology & Xinjiang Production & Construction Corps Key Laboratory of Protection and Utilization of Biological Resources in Tarim Basin, Tarim University, Alar 843300, China; 2 State Key Laboratory of Microbial Metabolism, School of Life Sciences and Biotechnology, Shanghai Jiao Tong University, Shanghai 200240, China

**Keywords:** *Lentzea*, biosynthetic gene cluster, comparative genomics, mobile genetic elements

## Abstract

**Background/Objectives:** Rare actinomycetes are considered underexplored reservoirs of bioactive natural products, yet genus-level evolutionary processes underlying diversity and novelty in specialized metabolite biosynthetic potential remain poorly understood. **Methods:** We analyzed 652 high-quality, non-redundant *Pseudonocardiaceae* genomes, including 59 *Lentzea* genomes, using phylogenomics, pangenome analysis, antiSMASH, BiG-SCAPE/MIBiG comparisons, orthogroup-informed BGC conservation analysis, and five genomic context criteria for MGE association. **Results:** Phylogenomics confirmed the monophyly of *Lentzea* spp. and resolved six intrageneric clades. Pangenome analysis revealed an open architecture with core–accessory functional differentiation. Comparative genome mining revealed a rich BGC repertoire in *Lentzea* spp., with no significant correlation between BGC abundance and genome size. Family-scale BiG-SCAPE clustering identified numerous *Lentzea*-specific, putatively novel GCFs in RiPP, terpene, and NRPS classes. Six lineage-associated BGC groups retained conserved biosynthetic backbones alongside local gene content and domain architecture variation, whereas MGE-associated BGCs were enriched among lower-prevalence GCFs. Sensitivity analyses supported conservation and mobility association patterns. **Conclusions:** Findings support a dual-pattern model of BGC conservation and mobility-associated diversification in *Lentzea*, providing complementary criteria for prioritizing biosynthetic candidates and a practical framework for genome-guided natural product discovery in rare actinomycetes.

## 1. Introduction

Actinomycetes have remained the predominant resource for the discovery of bioactive natural products with novel chemical scaffolds and clinically valuable drugs owing to their unrivalled metabolic versatility [1]. With the increase in diverse antibiotic resistance and the dramatic decrease in the success of traditional drug development, new bioactive natural product discovery remains an important priority. Advances in genome sequencing have revealed a vast uncharacterized reservoir of biosynthetic gene clusters (BGCs) for natural product discovery in various microbial genomes, which have revolutionized the natural product discovery methodology through bioinformatics tools. Bioinformatics tools for exploring novel BGCs in public databases integrate large-scale genomic data with phylogenetic analyses and sequence-similarity network analysis. Specifically, genome mining platforms such as antiSMASH [2], PRISM [3] and DeepBGC [4] have enabled systematic prediction of biosynthetic gene clusters from microbial genomes, while comparative frameworks such as BiG-SCAPE [5], BiG-SLiCE [6] and BiG-FAM [7] organize BGCs into sequence similarity networks and gene cluster families. Together with curated reference resources such as MIBiG [8], these tools have substantially accelerated natural product discovery. Moreover, the research paradigm has shifted from chemical or bioactivity phenotype-driven discovery to data-guided genetic mining and bioactivity prediction analysis, systematically expanding the analytical scope from individual genomes to the pangenome level [9,10,11].

Meanwhile, to discover novel bioactive natural compounds, researchers have expanded the search for microbial resources to extreme or unusual environments (e.g., marine sediments, desert lithosols, plant endophytes, mangroves, caves) that are rich in unusual specialized BGCs to enable survival under challenging environments [12,13,14]. For example, microorganisms isolated from acid mine environments and other extreme habitats have yielded azaphilone-type metabolites with anti-inflammatory activity, including berkchaetoazaphilone B, which inhibited the production of pro-inflammatory cytokines [12]; different halophilic actinomycetes have produced actinopolysporins, tubercidin, actinopolysporins A–C and the antineoplastic antibiotic tubercidin, which stabilized the tumor suppressor Pdcd4 in a cell-based assay [13]; and the marine-derived MAR4 *Streptomyces* lineage is enriched in hybrid isoprenoid pathways that generate diverse meroterpenoid-type natural products [15]. As the research into microbial natural product discovery expands, the rare actinomycetes have appeared as the underexplored reservoirs of structurally diverse and biologically active natural products including antimicrobial (abyssomicin C from marine strain *Verrucosispora maris* AB-18-032) [16], antitumor (ansamitocin P-3 from *Actinosynnema pretiosum* ATCC 31565) [17], enzyme-inhibitory (acarbose from *Actinoplanes* sp. SE50/110) [18] and potent immunosuppressive activity (brasilicardin A from *Nocardia brasiliensis* IFM 0406) [19,20,21,22].

Among the rare actinomycetes, the *Lentzea* genus of the family *Pseudonocardiaceae* is widely distributed in diverse soils, rhizospheres of plants, and extreme environments. In fact, members of this genus have been isolated from an unusually wide spectrum of niches, including arid desert [23,24], acidic soils [25], high-altitude plateaus [26,27], and oligotrophic karst caves [28], pointing to pronounced ecological versatility. Recent investigations have revealed that this genus possessed remarkable abilities to produce new diverse secondary metabolites. From various strains, many novel compounds with notable activities were obtained, including cycloimidamicin A, which exhibits potent antibacterial activity against Gram-negative bacteria [29]; Lentzeacin A, which has antitumor, anti-diabetic, and antibacterial properties [30]; the heterodimeric cyclopeptide petrichorin A, with potent antibacterial activity; and a series of new sesterterpenes derived from two multiproduct synthases [31,32]. These findings highlight that *Lentzea* spp. harbors significant biosynthetic potential for the discovery of bioactive new natural products. However, the systematic characterization of the genus-level pangenome of *Lentzea* spp., the diversity of their BGCs, the members of genus-specific BGCs, and the factors contributing to the novelty of BGC still remained elusive.

**Figure 1:**
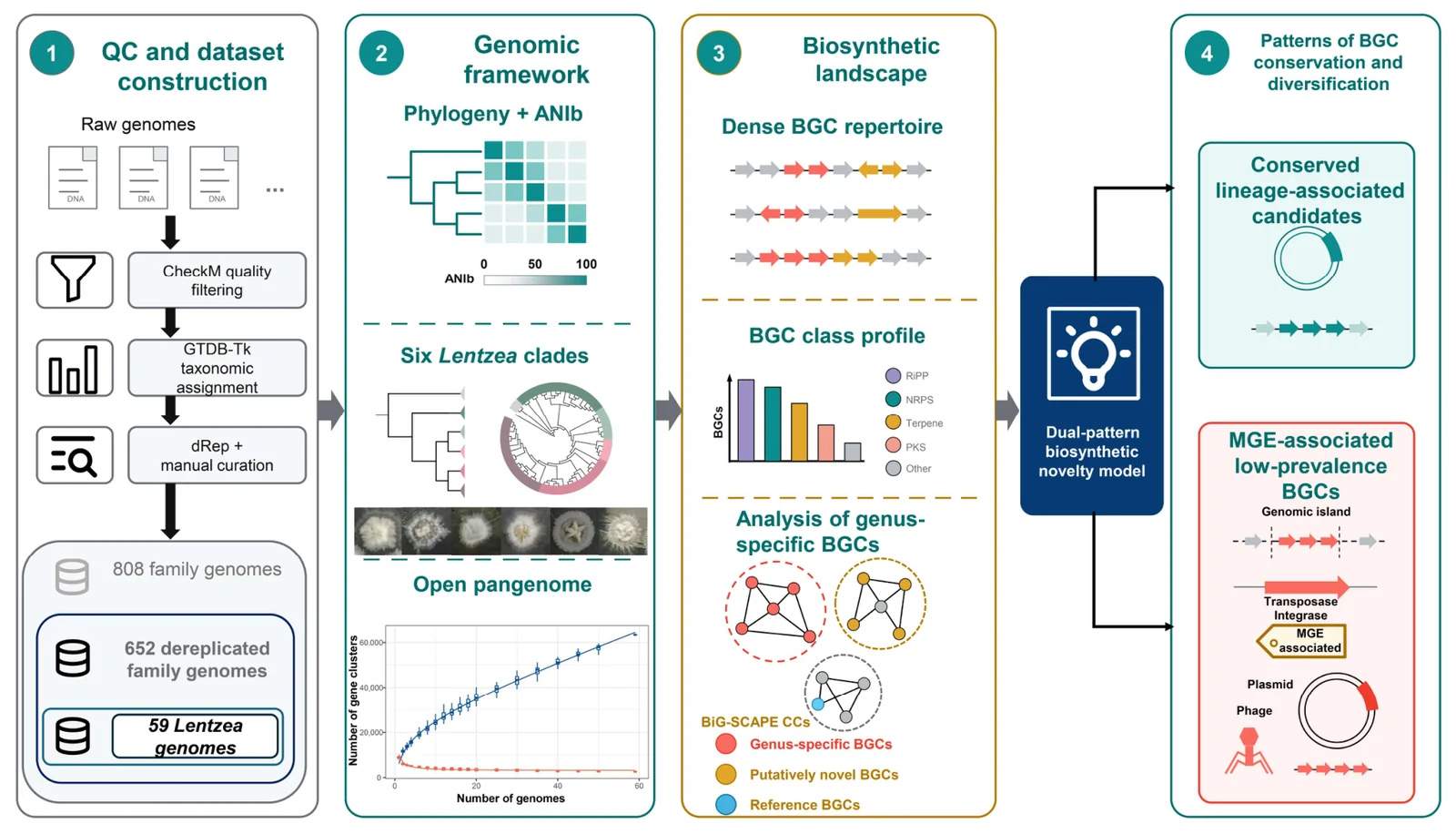
Overall analytical scheme for the *Lentzea* comparative genomics and biosynthetic gene cluster study. The workflow links quality control and dataset construction, phylogenomics and ANIb, pangenome analysis, family- and genus-scale BGC profiling, conservation-tier stratification, prioritization of lineage-associated BGC candidates, integration of MGE-associated evidence, and phylogeny- and mobilome-guided genome mining priorities.

To systematically elucidate the phylogeny and decipher the cryptic biosynthetic potential of specialized metabolites in rare *Lentzea* species, we developed an integrative comparative genomic and genome mining framework bioinformatics analysis pipeline across 652 *Pseudonocardiaceae* genomes, including 59 high-quality *Lentzea* genomes (Figure 1). We found that *Lentzea* spp. form a well-supported monophyletic lineage within *Pseudonocardiaceae* and contain an open pangenome with substantial accessory genome content. Comparative genome mining underscored the taxonomically distinct and rich BGC repertoire of *Lentzea* spp. within its family. Integrating BGC conservation with mobility-associated genomic contexts revealed two complementary patterns of specialized metabolic diversity in this genus. This framework provides a practical basis for prioritizing *Lentzea* strains and BGCs for natural product discovery: conserved, lineage-associated BGC groups lacking linked MIBiG references can be selected as candidates for genus signature chemistry, whereas rare or accessory BGCs associated with mobility-related signals can guide strain-level discovery of biosynthetic diversity.

## 2. Results

### 2.1. Phylogenomic Analysis Reveals the Monophyletic Origin and Distinct Intra-Genus Lineages of the Lentzea Genus

**Figure 2:**
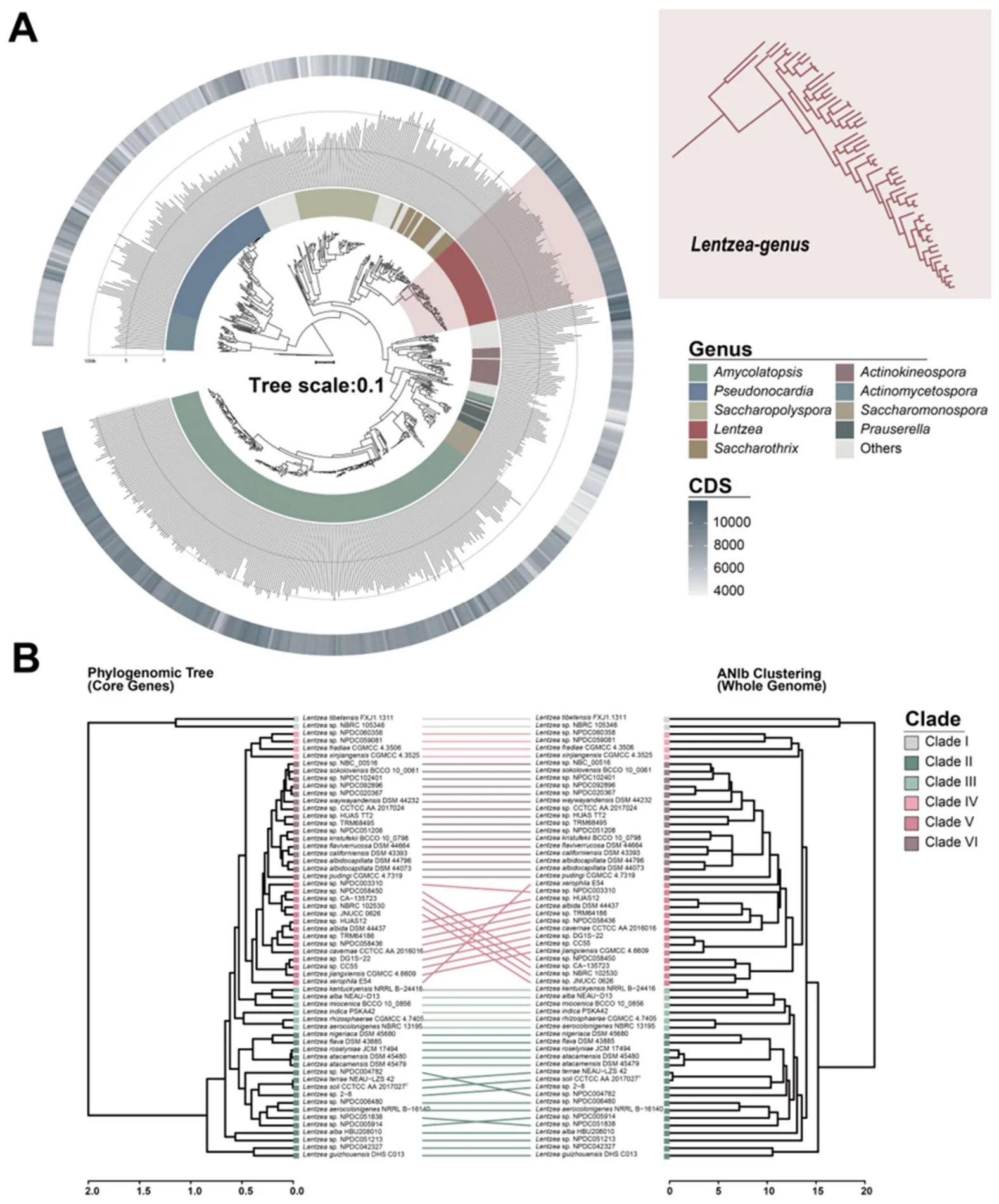
The genome-driven phylogenomic delineation of *Lentzea* spp. within *Pseudonocardiaceae*. (**A**) Family-scale phylogenomic tree of the dereplicated *Pseudonocardiaceae* genomes, with major genera color-coded in the inner ring and CDS abundance as the outer track; the inset highlights the *Lentzea* lineage used for subsequent genus-level analyses. (**B**) Tanglegram linking the core gene phylogenomic tree and whole-genome ANIb clustering for the 59 retained *Lentzea* genomes, with connecting lines for identical genomes and tip colors denoting six phylogenetic clades; scale bars are shown for both trees.

To systematically understand the characteristics of phylogenesis and potentiality of secondary metabolism for the genus *Lentzea* within the family of *Pseudonocardiaceae*, we isolated four strains of the *Lentzea* genus from samples from different environments and combined them with three previously preserved strains of the *Lentzea* genus to obtain a total of seven pure culture strains of the *Lentzea* genus. Their chromosomes were sequenced with Illumina and Nanopore technologies, depositing in GenBank of NCBI (PRJNA1496027). All their genome sequences revealed circular chromosomes of 8.8 to 10.5 Mbp, the G+C content ranged between 68.20% and 69.91%, and there were 8583 to 10,118 protein-coding genes, which were quite consistent with the other public strains for this genus. Meanwhile, we collected another 874 genomic sequences of the family of *Pseudonocardiaceae* from Refseq, including 61 publicly available strains of *Lentzea*. All obtained genome sequences underwent quality control and dereplication procedures, ultimately yielding a total of 652 *Pseudonocardiaceae* genomic sequences for further phylogenetic analysis, in which 59 genomes of the strains from *Lentzea* were obtained (Figure S1, Table S2). Based on the concatenated alignment of the 138 housekeeping genes from actinobacteria, we constructed a phylogenetic tree (Figure 2A) that comprised 652 genomes of *Pseudonocardiaceae* strains and three genomes from the outgroup strains through the workflow of GToTree v1.8.16. Referring to the phylogenetic positions of the typical strains from closely related families within the order of *Corynebacteriales* as the outgroups, the rooting strategy clearly showed an evolutionary trend within the family of *Pseudonocardiaceae*. The divergence distance between outgroups and ingroups aligned well with the established taxonomic relationships. All 59 strains of the *Lentzea* genus clustered in a monophyletic clad and neighbored the strains of genera *Saccharothrix* and *Actinosynnema*. The branch node received the perfect SH-like evidence (Support value = 1.00), strongly exhibiting *Lentzea* as a distinct genus within the family of *Pseudonocardiaceae*. Members of the genus *Lentzea* exhibited intrageneric conservation in genome size and coding sequence (CDS) number (outer ring of Figure 2A), whereas their sister groups (such as *Amycolatopsis*, *Saccharopolyspora* and *Pseudonocardia*, etc.) displayed divergent distribution patterns; this result further suggested their specific evolutionary trajectory.

To understand the evolutionary relationships among the members within the genus *Lentzea*, we carried out a high-resolution phylogenomic reconstruction based on the analysis of orthologous genes. OrthoFinder analysis of the 59 strains of the genus *Lentzea* identified 2873 orthologous gene families. Of these, 1719 single-copy orthologous groups were selected, and the corresponding protein sequences were used to construct a phylogenetic tree for the genus *Lentzea*. *Saccharothrix espanaensis* DSM 44229 was used as the outgroup, and the best-fit substitution model was selected using ModelFinder. As a result, the selected Q.INSECT+F+R9 models exhibited the lowest Bayesian Information Criterion (BIC) score (BIC = 15,630,872.467) and exceptional reliability, which clearly delineated the evolutionary relationships among strains of the *Lentzea* genus from the maximum likelihood tree. Isolation sources were distributed across multiple phylogenetic positions rather than defining a single habitat-restricted lineage (Figure S2). To assess the phylogenetic congruence between core genes and genome-wide profiles, we constructed a tanglegram to compare the phylogenomic tree from 1719 single-copy core genes with the hierarchical clustering dendrogram derived from the whole-genome average nucleotide identity (ANIb). Based on the topology analysis of the phylogenetic tree, the 59 *Lentzea* strains were classified into six major clades (Clade I to VI). The major intrageneric clades were also recovered under the alternative LG model, indicating that the principal *Lentzea* topology was not driven solely by the selected Q.INSECT substitution model (Figure S3). Comparison of the core protein phylogeny with ANIb-based whole-genome clustering showed broad concordance in Clades I, II, III, IV, and VI, whereas several strains in Clade V showed topological discordance between the two representations. In a word, through integrative phylogenomic approaches, we demonstrate the convinced monophyly origin of the *Lentzea* genus, reconstructing its microevolutionary patterns to establish an evolutionary framework for this phylogenetically unique clade.

### 2.2. The Open Pangenome of Lentzea spp. Exhibits Functional Divergence with Core and Accessory Genes

To evaluate the genomic plasticity and functional gene distribution patterns within the genome of the *Lentzea* strains, we performed a comprehensive pangenome analysis with the IPGA pipeline [33]. This pipeline integrated multiple pangenome analysis tools (including Roary, Panaroo, OrthoFinder, etc.) to determine the reliability of the output results. Using the IPGA-selected OrthoFinder profile, we generated the pangenome and core genome accumulation curves for the 59 *Lentzea* genomes.

**Figure 3:**
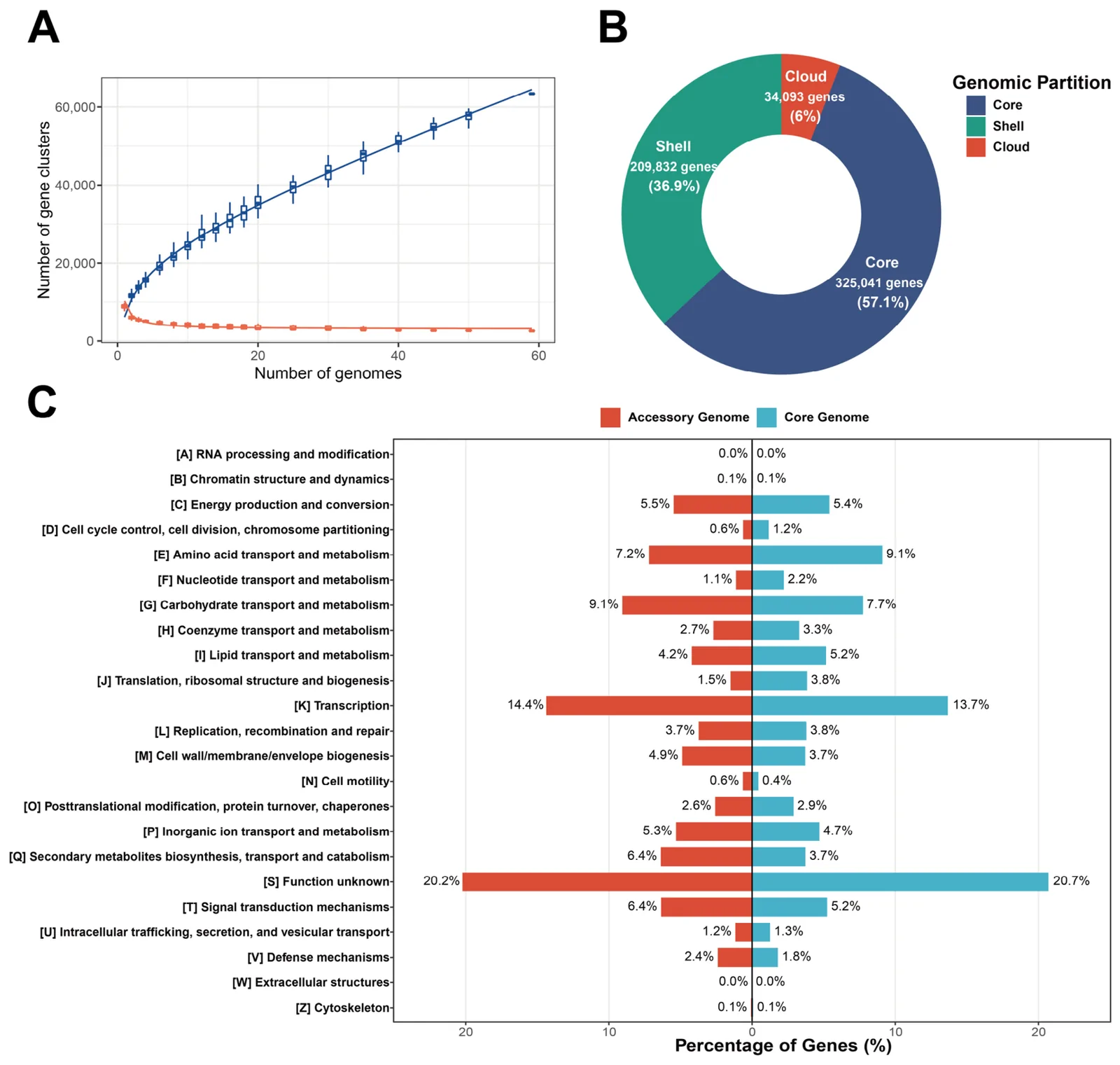
The pangenome of *Lentzea* spp. is open and functionally divided into core and accessory compartments. (**A**) For the *Lentzea* genome set, the pangenome accumulation curve rises with each additional genome, indicating ongoing gene discovery, while the core genome decay curve declines, reflecting progressive refinement of shared genes. (**B**) Gene count-based composition of the *Lentzea* pangenome, partitioned into core, shell, and cloud genomic compartments. (**C**) Functional distribution of core and accessory genes across COG categories. Percentages are calculated within each genomic partition, allowing for comparison of functional biases between the conserved core genome and the more variable accessory genome.

Pangenome reconstruction of the 59 *Lentzea* genomes revealed an open genomic architecture (Figure 3A). The core genome curve declined rapidly and approached an asymptote, whereas the pangenome curve continued to increase with additional genomes, consistent with ongoing gene gain and loss within the genus. The continued expansion of the pangenome curve is consistent with an open pangenome, a common feature of bacterial groups in which gene gain, gene loss and ecological diversification shape strain-level gene repertoires [34,35]. Furthermore, based on OrthoFinder annotation, 3132 core orthogroups were discovered from the 59 genomes of *Lentzea* strains. Using 744 non-*Lentzea* genomes from the pre-dRep family dataset (808 genomes, including 64 *Lentzea*) as background, a database comprising 5.66 million proteins was constructed. Then, using the sequences of the 3132 core orthogroups to the BLAST v2.5.0 search within the database, we identified 47 core orthogroups that were unique to the *Lentzea* genus. This finding strongly supported the definition of the genetic basis of this genus.

Using prevalence-based classification, we separated orthogroups into core, shell and cloud compartments (Figure 3B). The pangenome architecture analysis of *Lentzea* genus revealed that the core genes contained 57.1% of the total gene repertoire (325,041 genes), while the accessory genome, comprising shell (209,832 genes, 36.9%) and cloud (34,093 genes, 6%), still possessed >40% of the genetic content (Table S4). Functional annotation through COGs revealed a functional specialization between the core genome and the accessory genome areas (Figure 3C). The core genome was significantly enriched in categories essential for fundamental cellular processes, including translation, ribosomal structure, and biogenesis ([J], 3.8%); amino acid transport and metabolism ([E], 9.1%); and energy production and conversion ([C], 5.4%), whereas the accessory genome exhibited a preference of adaptation. A notable distinction was observed in the natural product biosynthesis genes, transport, and regulators of secondary metabolites ([Q] and [K]), in which nearly twice the number of members appeared in the accessory genome (6.4%) compared to the core genome (3.7%). Remarkably, the category of function unknown genes ([S]) exhibited the highest proportion in both the core (20.7%) and accessory (20.2%) genomes, which highlighted the vast reservoir of uncharacterized genetic “dark matter” within the *Lentzea* genus. Finally, these results exhibited the housekeeping functions of the core genes ensured the basic survival abilities of such species in this genus to endure various habitats. The encoding potential of the niche adaptation and metabolic diversity within this genus primarily occurred in non-core genes.

Functional annotation further indicated a clear division between conserved cellular functions and accessory adaptive potential. Core orthogroups were enriched in housekeeping categories such as translation, amino acid metabolism and energy production, whereas accessory orthogroups showed higher representation of transcriptional regulation and secondary metabolism-related functions (Table S5). The high proportion of genes with unknown function in both compartments highlights an additional reservoir of uncharacterized biology in *Lentzea* spp.

### 2.3. Lentzea spp. Harbor an Extensive and Diverse Reservoir of Cryptic BGCs for Secondary Metabolites Within Pseudonocardiaceae

**Figure 4:**
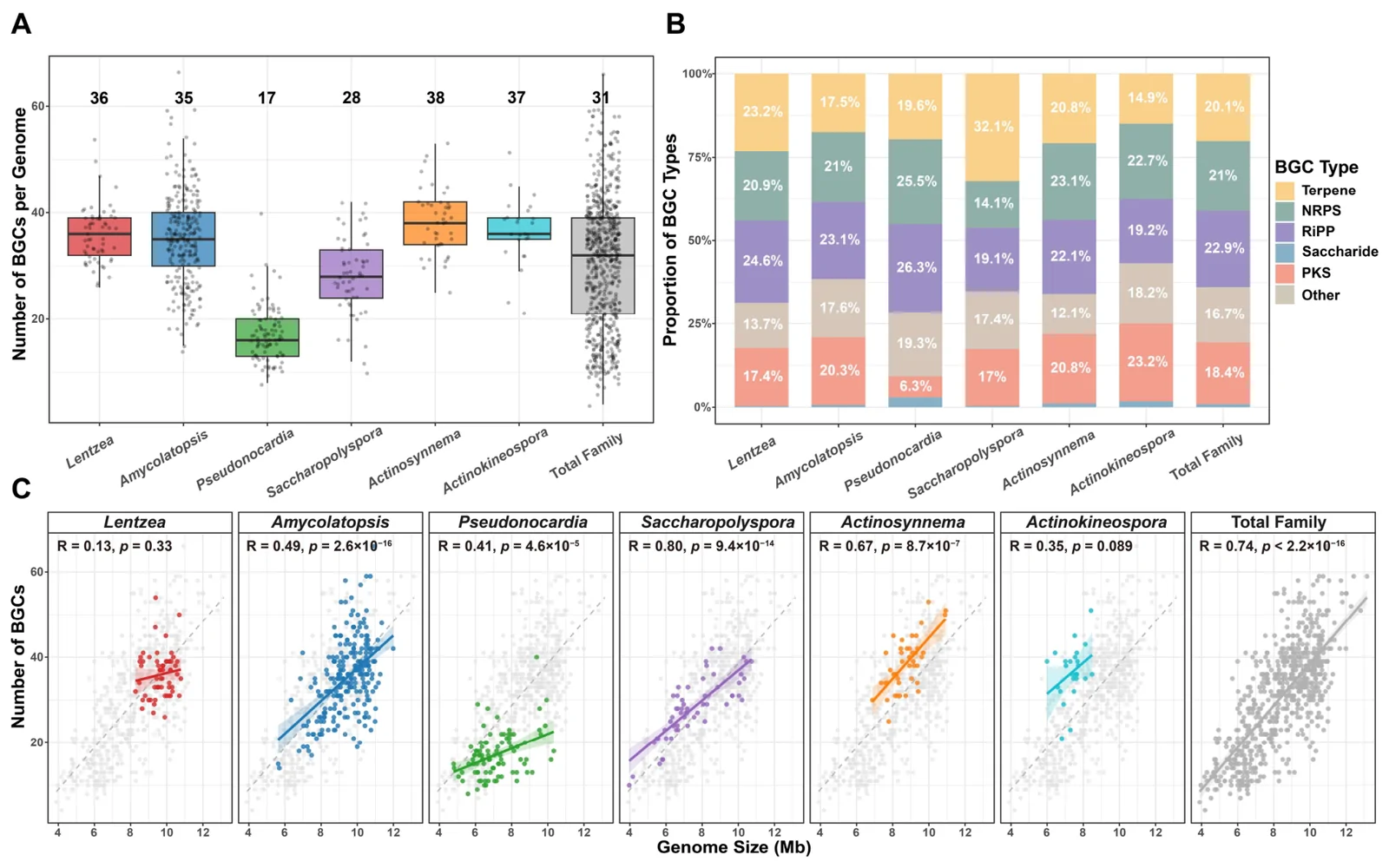
*Lentzea* spp. harbor an abundant and taxonomically distinctive BGC repertoire within *Pseudonocardiaceae*. (**A**) Per-genome BGC abundance across genera and the total family dataset. Boxes summarize the distribution of BGC counts per genome, with individual genomes shown as points. (**B**) Relative composition of BGC classes across *Lentzea* spp., the five most represented background genera (*Amycolatopsis*, *Pseudonocardia*, *Saccharopolyspora*, *Actinosynnema* and *Actinokineospora*), and the full dereplicated family dataset. BGC classes were grouped into NRPS, PKS, RiPP, terpene, saccharide, and other categories. (**C**) Relationship between genome size and BGC count within each genus. Grey points indicate the global *Pseudonocardiaceae* background, colored points indicate the focal genus in each panel, and fitted lines summarize genus-specific trends.

To systematically assess the secondary metabolic potential of *Lentzea* genus within the family of *Pseudonocardiaceae*, we conducted a whole family-scale comparative genomic analysis of the biosynthetic gene clusters (BGCs) for the composition and abundance features with antiSMASH (Table S6). The results showed that each strain harbored an average of about 31 biosynthetic gene clusters (BGCs) in the family of *Pseudonocardiaceae* (Figure 4A). Notably, the 59 *Lentzea* genomes harbored a mean of 35.93 BGCs per genome and had a mean genome size of 9.65 Mb (Table S9). *Lentzea*, *Actinosynnema* (about 38 BGCs), *Amycolatopsis* (about 35 BGCs) and *Actinokineospora* (about 37 BGCs) comprised the high-BGC cohorts within the *Pseudonocardiaceae* family (Table S9). Our BGC repertoire analysis further observed that *Lentzea* spp. displayed a considerable different type of BGCs, which suggested the structural diversification encoding capacities in secondary metabolites (Figure 4B). Moreover, the *Lentzea* strains maintained the substantial proportions of ribosomally synthesized and post-translationally modified peptides (RiPPs, 24.6%), nonribosomal peptide synthetases (NRPSs, 20.9%), and terpenes (23.2%), whereas Actinosynnema harbored a significant preference for polyketide synthases (PKSs, 32.1%), and *Saccharopolyspora* was shown to be rich in terpenoids (Terpene, 32.1%). These characteristics of BGCs in *Lentzea* strains suggested that they may have a unique biosynthetic niche with significant bioprospecting potential for new peptide- or terpene-derived and hybrid natural products.

Generally, a sufficiently large genome of Actinobacteria was the prerequisite for accommodating the rich diverse of secondary metabolite BGCs. At the *Pseudonocardiaceae* family level, genome size exhibited a highly positive correlation (R = 0.74, *p* < 2.2 × 10^−16^) with BGC abundance, consistent with broad surveys showing that larger bacterial genomes, especially those of actinomycetes, often encode more specialized metabolism capacity (Figure 4C) [36]. The similar pattern also observed in the genera of *Saccharopolyspora* and *Actinosynnema*. However, the substantial BGC abundance in *Lentzea* spp. showed no significant linear correlation (R = 0.13, *p* = 0.33) between genome size and BGC number, indicating that the genus is better described as maintaining elevated BGC density rather than simply expanding genome size (Table S10). Detailed analysis of the BGC counts for individual genome showed that even *Lentzea* strains with smaller genomes (~8–10 Mb) still maintained a higher number of BGCs (approximately 30–40), exceeding the *Pseudonocardiaceae* family-wide average BGC count. This pattern indicates that *Lentzea* maintains a substantial BGC repertoire across the observed range of genome sizes. Such a high BGC density might boost the chemical defense capabilities and competitive advantages in challenging ecological niches, which also offers a promising host for discovery of structurally novel bioactive natural products. Similar high-density and taxonomically distinctive BGC repertoires have been reported from underexplored rare actinomycete lineages, supporting the value of rare actinomycete-focused genome mining [21,22].

### 2.4. Lentzea spp. Encode a Genus-Specific Repertoire of Diverse and Novel BGCs

**Figure 5:**
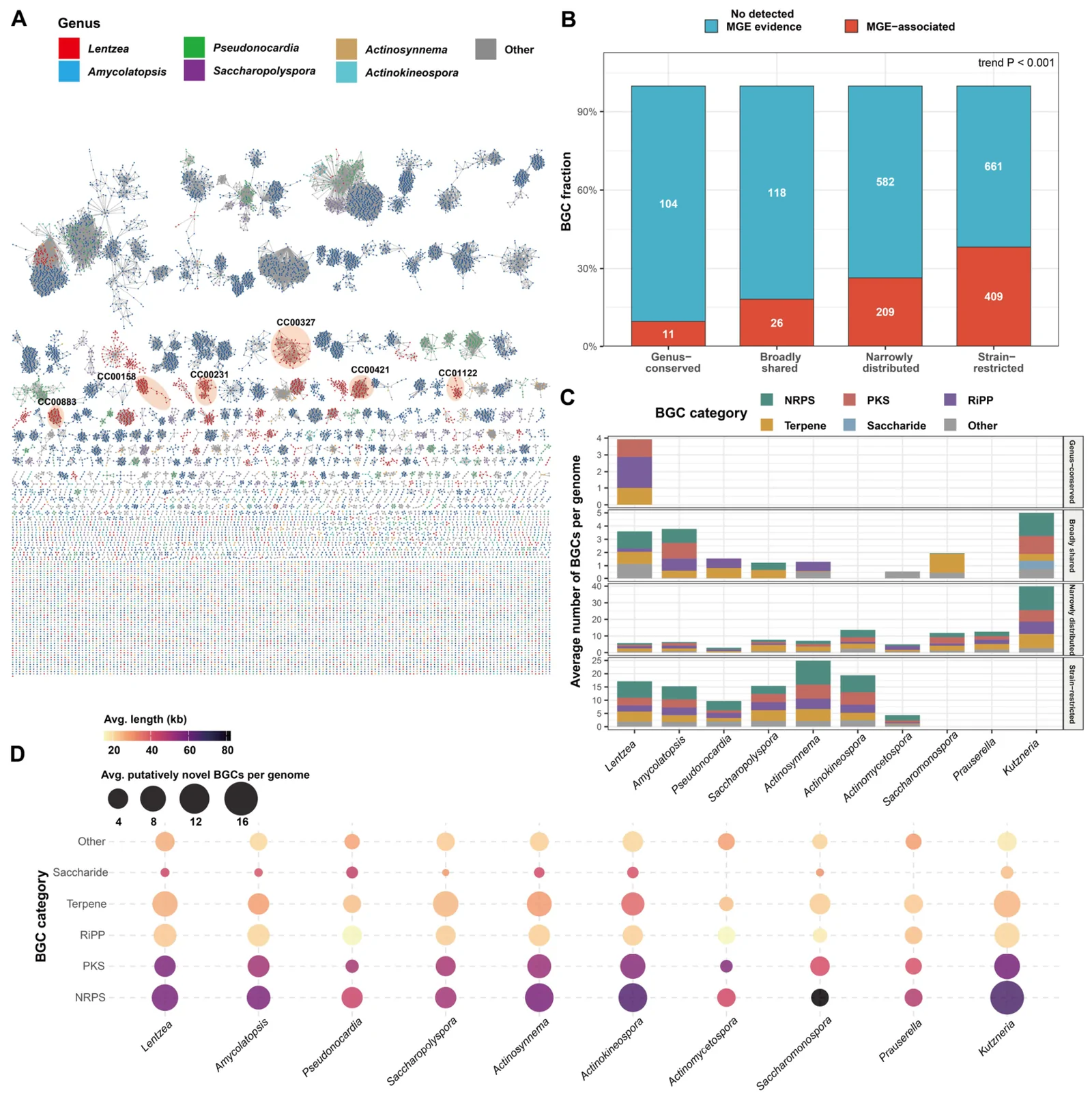
Family-scale BGC similarity analysis reveals genus-restricted and conservation-tiered biosynthetic diversity. (**A**) BiG-SCAPE BGC similarity network for the *Pseudonocardiaceae* dataset at cutoff 0.20. Nodes represent BGCs and are colored by genus; edges represent BiG-SCAPE similarity relationships. The network highlights the separation of many genus-associated BGC connected components. (**B**) Proportions of BGCs without detected MGE-context evidence and MGE-associated BGCs across four GCF-based conservation tiers in *Lentzea* spp. Tiers are defined by the prevalence of the corresponding GCF among the 59 *Lentzea* genomes: genus-conserved, broadly shared, narrowly distributed, and strain-restricted. Numbers inside bars indicate BGC counts, and the trend test evaluates the ordered change in the MGE-associated fraction across tiers. (**C**) Average per-genome numbers of genus-specific BGCs across major genera. Genus-specific BGCs were selected from single-genus CCs at cutoff 0.20 and stratified by within-genus CC prevalence and BGC category. (**D**) Distribution of putatively novel BGCs within this CC-defined genus-specific subset. Dot size represents the average number of these BGCs per genome, and color represents their mean BGC length.

To comprehensively evaluate the diversity of BGCs for secondary metabolites in the *Pseudonocardiaceae* family, we constructed a sequence similarity network through BiG-SCAPE comprising all the BGCs from the strains within the family (Figure 5A, Figure S5). The BGCs from the entire family clustered into the Gene Cluster Family (GCF) networks. Grouping homologous BGCs into GCFs provides a practical framework for dereplication, taxonomic comparison and prioritization of biosynthetic diversity across large genome collections [7,37]. Across the tested cutoffs of 0.10–0.30, a cutoff of 0.20 retained a high proportion of *Lentzea*-specific CCs while limiting mixed genus clustering and excessive merging into the largest component (Figure S4, Table S11). We therefore used 0.20 for the principal network analyses and treated the remaining cutoffs as sensitivity analyses. The prevalence of a BiG-SCAPE GCF within the genus defines four conservation tiers: genus-conserved, broadly shared, narrowly distributed, and strain-restricted (Figure 5B). Although certain GCFs are distributed throughout multiple genera, many GCFs still displayed genus-specific enrichment patterns. It was interesting that *Lentzea* BGCs formed multiple independent, tightly connected components within the network, with limited connections to BGCs from other genera. This finding presented that the strain from *Lentzea* genus harbored a rich repertoire of evolutionarily specific BGCs, constituting a species-specific secondary metabolic “BGCs-encoding fingerprint” that was consistent with its phylogenetic position in the family of *Pseudonocardiaceae* (Table S12). To reduce the cumulative bias arising from extensive sequencing efforts in genera such as *Amycolatopsis* and *Pseudonocardia*, we further introduced genome-normalized metrics to characterize a specialized BGC landscape per genus. For the genus-specific repertoire analysis, BGCs were selected from single-genus CCs and stratified by the within-genus prevalence of their CCs (Figure 5C; Table S12). Within the genus-specialized BGC repertoire of the *Lentzea* genus, RiPPs and NRPSs constituted the predominant members (Figure 5C).

To address the novelty concern of the BGCs in natural product discovery, we further estimated both the number of unidentified BGCs (represented by bubble size) and the length of DNA sequence for BGCs (indicated by bubble color) through visualization bubble plots, which clearly presented the numbers and ratio of the new BGCs across different genera in the *Pseudonocardiaceae* family (Figure 5D). Because an absence of close MIBiG matches indicates database distance rather than direct chemical characterization, we refer to these clusters as putatively novel BGCs [7]. The *Lentzea* genus prioritized the BGCs of terpenes, NRPSs and RiPPs flagged by large bubble size, which revealed a high density of novel BGCs within the genomes, and the uncharacterized BGCs represented the cryptic “dark matter” from the MIBiG database (Tables S7 and S8). Moreover, the BGCs of PKSs and NRPSs from the *Lentzea* genus featured a relatively long average DNA sequence length (>60 kb). This result suggested that these BGCs might encode structurally sophisticated or varied modified new natural products. In a word, our data exhibited that the genomes of *Lentzea* spp. not only harbored abundant genus-specialized BGCs, but also showed the enrichment in terpenes, RiPPs and NRPSs, especially displaying RiPPs as potential targets for mining new natural products (Table S13). In our family-scale network, the genus-skewed distribution of BGC classes and CC-level genus-restricted components further suggests that biosynthetic diversity is partly organized by lineage history, while providing a basis for evaluating lineage-associated BGC conservation alongside mobility-associated diversification in *Lentzea* spp. (Figure S6, Table S12).

### 2.5. Lineage-Associated BGC Groups Retain Conserved Biosynthetic Backbones in Lentzea spp.

**Figure 6:**
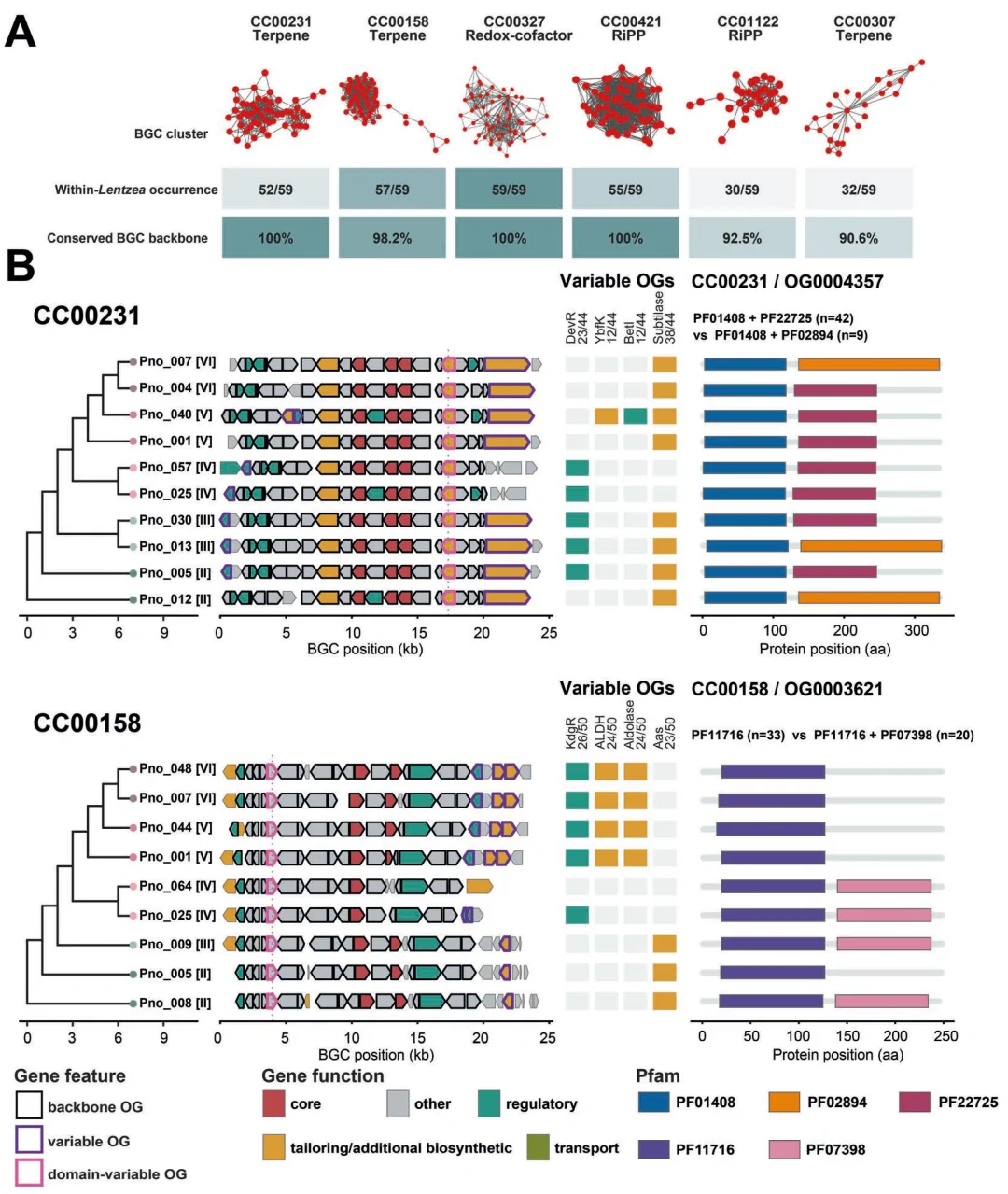
Prioritization and characterization of six lineage-associated BGC groups in *Lentzea* spp. (**A**) Overview of six prioritized connected components (CCs), ordered as CC00231, CC00158, CC00327, CC00421, CC01122, and CC00307. The top row shows the BGC similarity network for each prioritized CC. Within-genus occurrence indicates the number of the 59 focal *Lentzea* genomes carrying each CC. Conserved BGC backbone shows the mean recurrence of consensus core biosynthetic orthogroups (OGs) among CC members. All six candidates were *Lentzea*-restricted in the 652-genome BiG-SCAPE c0.20 analysis. (**B**) Representative comparisons of CC00231 and CC00158. BGCs were aligned to the domain-variable OG. Gene fill denotes the antiSMASH-derived functional category. Black, purple, and magenta solid outlines indicate backbone OGs recurring in at least 90% of non-edge-complete BGCs, selected variable OGs, and the domain-variable OG, respectively. Adjacent matrices show variable OG presence. Right subpanels display Pfam architectures of proteins assigned to the domain-variable OG, using accession-specific colors. Representative BGCs are shown; frequencies and denominators were calculated from all evaluable CC members.

Lineage-associated BGC groups can retain conserved biosynthetic backbone components while exhibiting local gene content variation, sequence divergence, or boundary changes. Hence, they can be served as a valuable information for elucidating their contribution to the diversity and novelty of BGCs within the genus *Lentzea*. To identify BGC lineages compatible with long-term retention within *Lentzea* spp., we reprioritized BiG-SCAPE c0.20 connected components (CCs) using an OrthoFinder-informed conservation approach. Candidate status was based primarily on recurrence across the 59 core genomes and on the recurrence of consensus core biosynthetic orthogroups (OGs) among CC members. We then evaluated family-scale taxonomic restriction across the 652-genome dataset, while region completeness and MGE evidence were retained as orthogonal quality checks (Supplementary Figure S7 and Data S4, Table S14). This screen prioritized six lineage-associated CCs: four genus-scale candidates (CC00327, CC00421, CC00158, and CC00231) and two clade-associated candidates (CC00307 and CC01122). The four genus-scale CCs occurred in 59, 55, 57, and 52 of the 59 *Lentzea* genomes, respectively, with consensus core OG recurrences ranging from 98.2% to 100% (Figure 6A). Under the current family-scale sampling and BiG-SCAPE c0.20 mapping, all four were *Lentzea*-restricted, with no non-*Lentzea* BGC members and no linked MIBiG records.

Gene content analysis showed that backbone conservation coexisted with recurrent differences in regulatory and tailoring/additional biosynthetic OGs. Representative BGCs aligned by the domain-variable OG retained a shared gene order while differing in selected local OGs (Figure 6B; Supplementary Figure S8; Data S4, Table S15). In CC00231, DevR, YbfK, BetI, and Subtilase OGs occurred in 23/44, 12/44, 12/44, and 38/44 non-edge-complete BGCs, respectively. In CC00158, KdgR, ALDH, Aldolase, and Aas, OGs occurred in 26/50, 24/50, 24/50, and 23/50 complete BGCs, respectively. These patterns concentrate variation in regulatory and enzyme-associated genes around conserved BGC scaffolds.

CC00231 also contained lineage-structured domain variation within a homologous OG. This terpene CC occurred in 52 genomes and showed 100% mean consensus core OG recurrence. Among 51 proteins assigned to the tailoring/additional biosynthetic OG0004357, 42 contained PF01408 followed by PF22725, and nine contained PF01408 followed by PF02894 (Figure 6B; Data S4, Table S15). The two architectures required six parsimony transitions on the species tree, compared with a median of nine after label permutation. The difference remained significant after Benjamini–Hochberg correction (q = 0.036), supporting phylogenetic clustering of the alternative C-terminal Pfam states (Data S4, Table S16).

CC00158 contained a second recurrent domain architecture within a highly conserved terpene scaffold. This CC occurred in 57 genomes and showed 98.2% mean consensus core OG recurrence. PF11716 was present in all 53 proteins assigned to OG0003621. Twenty proteins also contained PF07398, while 33 contained PF11716 alone (Figure 6B; Data S4, Table S15). Species tree mapping yielded 14 observed parsimony steps and a permutation median of 16 (adjusted q = 0.183). We therefore describe PF07398 as a candidate domain addition or alternative Pfam architecture. Across all six candidates, the same analysis retained 13 recurrent domain architecture events in five CCs, spanning regulatory, tailoring/additional biosynthetic, and other BGC genes (Data S4, Table S16). Together, these results show that recurrent and currently genus-restricted BGC lineages need not be architecturally invariant. Instead, highly conserved core OG backbones can coexist with repeated domain-level alternatives, consistent with intrageneric microevolution acting on retained biosynthetic scaffolds. In summary, the terpene BGCs in CC00231 and CC00158 retained conserved core biosynthetic backbones alongside variation in peripheral genes, a pattern compatible with long-term intrageneric retention [38,39].

### 2.6. Low-Prevalence BGCs in Lentzea spp. Are Enriched in Mobility-Associated Genomic Contexts

The bacterial mobilome comprises mobile genetic elements and mobility-associated loci, including genomic islands, plasmids, prophages or viral regions, transposon-, recombinase-associated and other mobility-related systems [40,41], that can remodel bacterial genomes and mediate the movement of adaptive traits across lineages [42,43]. We therefore considered mobilome association as an evidence-integrated genomic context and used it to assess whether accessory BGCs in *Lentzea* spp. are preferentially located in chromosomal regions exhibiting mobility signatures [2,44,45,46]. For each BGC, five mobility-related criteria were evaluated. First, we tested whether the BGC overlapped a predicted genomic island using genomic island calls as region-level evidence for atypical or recombination-prone genomic context [45]. Second, we searched for Prokka-annotated mobile element genes, including transposase-, integrase- or recombinase-related annotations, within the BGC or its 10 kb flanking regions [44]. Third, we screened antiSMASH SMCOG annotations within the BGC for mobile element-related functional categories [2]. Fourth and fifth, we tested whether the BGC overlapped geNomad-predicted plasmid regions or geNomad-predicted phage/viral regions, respectively [46]. BGCs containing at least one of the five criteria were classified as putative MGE-associated, while those meeting at least two criteria were further defined as a more stringent subset in robustness analyses. Among the 2120 *Lentzea* BGCs, 655 were associated with at least one MGE-evidence dimension, whereas 310 were supported by at least two dimensions and were retained as the stringent subset (Figure S9, Table S17).

**Figure 7:**
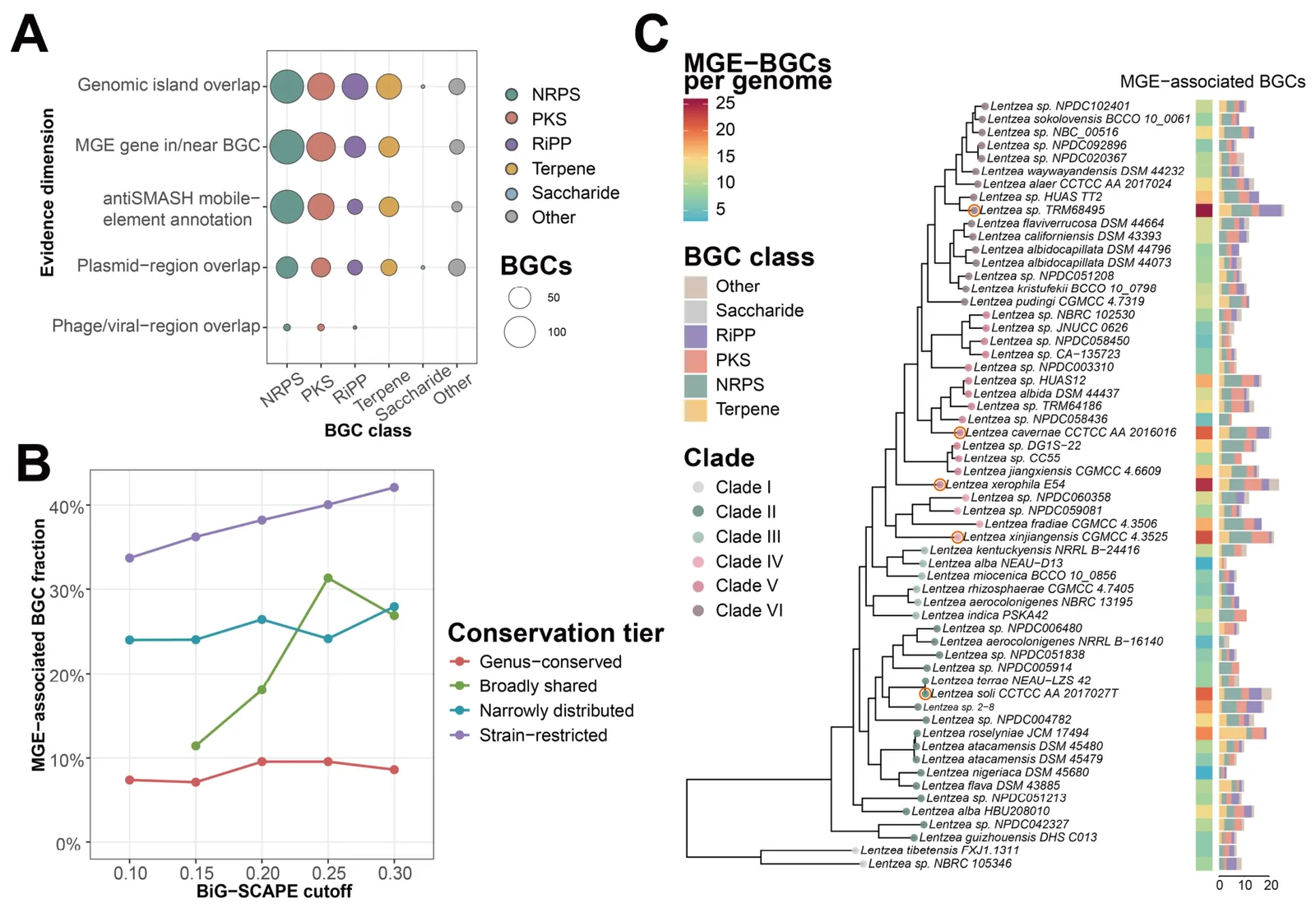
Mobilome-associated BGCs are linked to strain-level biosynthetic diversification in *Lentzea* spp. (**A**) Five-dimensional mobility evidence matrix across BGC classes. Bubble size indicates the number of BGCs supported by each evidence dimension, and color indicates BGC class. (**B**) Robustness of MGE-associated BGC enrichment across BiG-SCAPE cutoffs. Fractions of MGE-associated BGCs are shown for the four conservation tiers from cutoff 0.10 to 0.30. (**C**) Genome-level distribution of MGE-associated BGCs across the *Lentzea* phylogeny. The heat strip shows the number of MGE-associated BGCs per genome, and the stacked bars summarize their BGC-class composition. Highlighted genomes indicate strains with more than 20 MGE-associated BGCs.

The construction of an MGE evidence matrix revealed that MGE-associated BGCs were supported by multiple types of evidence and spanned various BGC classes, including NRPS, PKS, RiPP, terpene, and others (Figure 7A). MGE-associated BGCs were disproportionately represented in the lower-prevalence GCF tiers, and this trend was supported by the corresponding association and trend tests (Figure 5B, Table S18). In validating the robustness of BGC clustering in *Lentzea* spp. across multiple cutoffs (0.10, 0.15, 0.20, 0.25, 0.30), narrowly distributed and strain-restricted BGCs consistently displayed higher proportions of MGE associations than genus-conserved BGCs (Figure 7B, Table S19). We then mapped these loci onto the *Lentzea* phylogeny to identify genomes with high numbers of mobilome-associated BGCs (Figure 7C). More than 20 putatively MGE-associated BGCs were detected in several genomes, including *Lentzea* sp. TRM68495, *Lentzea xerophila* E54, *Lentzea xinjiangensis* CGMCC 4.3525, *Lentzea soli* CCTCC AA 2017027^T^ and *Lentzea cavernae* CCTCC AA 2016016. Several of these genomes occurred near the tips of local phylogenetic branches, suggesting that mobilome-associated BGC accumulation may contribute to fine-scale strain or species differentiation within *Lentzea* spp.

As an additional functional context analysis, we compared BGC-linked resistance- and transporter-related predictions from ARTS, CARD and TCDB and summarized their intersections and BGC-class distributions (Figure S10), indicating that biosynthetic loci can be spatially coupled with self-protection or efflux functions, with 470/655 (71.8%) carrying at least one candidate link within the BGC or its 10 kb flanking window, most commonly TCDB efflux/transporter annotations (419/655, 64.0%) (Table S20). Such coupling is consistent with the co-maintenance of BGCs and fitness-supporting functions in their host genomes. To summarize the count-based relationships among mobility state, BGC category and MIBiG context, we generated a descriptive alluvial representation of all 2120 *Lentzea* BGCs (Figure S11, Table S17). Overall, the enrichment of mobility-associated genomic contexts among low-prevalence BGCs complements the pattern of conserved, lineage-associated BGC groups and highlights strain-level biosynthetic diversity in *Lentzea* spp.

## 3. Discussion

The rare actinobacterial genus *Lentzea* (*Pseudonocardiaceae*) exhibits remarkable ecological versatility, colonizing extreme niches from arid deserts to karst caves, displaying a powerful adaptive ability to the complicated environment. As an underexplored resource of new bioactive natural product exploration, recent studies highlight their capacity for production of diverse novel bioactive secondary metabolites, underscoring their significant, untapped biosynthetic potential. To investigate the ecological niche, genus-level pangenome of *Lentzea* spp., and the features, novelty, and drivers of biosynthetic gene cluster (BGC) diversity, we integrated a *Pseudonocardiaceae*-scale comparative genomics approach with a *Lentzea*-focused pangenome analysis and characterized the coding potential of the BGCs. The analyzed *Lentzea* genomes, isolated from diverse ecological settings including soils, rhizospheres, and several extreme or oligotrophic habitats, exhibit an open pangenome, substantial accessory gene content, and a dense, taxonomically restricted BGC repertoire. The broad ecological distribution facilitates diverse pathways for their acquisition and integration of exogenous genetic material. The features of their open pangenome presents a vast untapped genetic repertoire in this population. Moreover, the 47 genus-specific orthogroups (OGs) identified herein define the genetic boundaries of the genus and can be developed as the genetic probes for targeted isolation and enrichment of *Lentzea* spp. from complex environmental samples.

The discordance between the core protein phylogeny and ANIb-based clustering in Clade V may reflect recombination, admixture, gene content change, or other sources of genomic discordance. Targeted recombination and synteny analyses, together with broader sampling, could help distinguish among these possibilities.

At the family level, genome size positively correlates with BGC number, consistent with the greater biosynthetic capacity typically observed in larger actinomycete genomes. Regarding the genetic potential for secondary metabolites, *Lentzea* spp. exhibited a moderate-to-high BGC abundance (Figure 4), reflecting the remarkable diversity and high taxonomic specificity of secondary metabolites among *Pseudonocardiaceae* strains. This observation may reflect differential retention and acquisition of biosynthetic loci during adaptation to heterogeneous habitats, where genome-encoded specialized metabolites may contribute to competition, stress tolerance, or niche persistence. Crucially, sequence similarity networks and novelty assessments (Figure 5) indicate that *Lentzea* spp. are significantly enriched in unique biosynthetic gene clusters (BGCs), highlighting its lineage-restricted potential for producing novel specialized metabolites. This finding aligns with the broader concerns that rare actinomycetes remain underexplored reservoirs of cryptic, taxonomically distinct biosynthetic pathways, thereby promoting the discovery of novel bioactive natural products.

Integrating genus-level GCF prevalence, family-scale CC context, MIBiG similarity, BGC synteny, and mobilome evidence, our analysis supports a dual-pattern model of BGC conservation and mobility-associated diversification in *Lentzea* spp. Conserved, lineage-associated BGC groups spanning multiple clades and lacking linked MIBiG references represent promising candidates for genus signature chemistry. The representative RiPP and terpene candidates illustrated that conserved BGCs maintain core backbones alongside variation in transport, regulatory, tailoring, or peripheral genes. In our analysis, CC00231 and CC00158 were prioritized as conserved terpene BGC groups. Such broadly conserved groups provide candidates for targeted activation to investigate the chemotaxonomic characteristics of *Lentzea* spp.

BGC conservation and MGE association represent distinct analytical dimensions that may overlap. Conserved lineage-associated groups are compatible with long-term intrageneric retention, including retention following early horizontal acquisition; the absence of detectable MGE context evidence does not exclude historical transfers whose signatures have been lost. Vertical retention, horizontal acquisition, gene loss, and recombination may all contribute to the observed patterns.

Alongside these conserved groups, strain-restricted and narrowly distributed BGCs were enriched in mobilome-associated signals. Such loci may be introduced, reshaped, or differentially retained through accessory genome turnover, thereby contributing to the biosynthetic diversification within the strain-specific BGCs. Furthermore, the co-localization of BGCs with transporters, efflux pumps, and resistance determinants (Figure S10) is consistent with the potential co-maintenance or co-mobilization of biosynthetic and self-protection functions. Such physical linkage may support host fitness through self-resistance and secretion and may facilitate the persistence of associated BGCs, including NRPS, PKS, RiPP, and related clusters.

At the genome level, this dual-pattern model aligns with pangenome theory, where conserved and accessory compartments reflect distinct patterns of inheritance, gain, loss, and ecological filtering [34,35,47]. At the BGC level, it is further supported by comparative studies revealing that biosynthetic loci can be shaped by vertical inheritance, horizontal exchange, recombination, and modular remodeling [39,48]. For *Lentzea* spp., integrating phylogenomic placement, GCF novelty, synteny conservation, mobilome context, and self-resistance/transporter linkages enables genome-guided discovery to evolve toward a more evolution-directed, targeted search for novel natural products. By integrating phylogenomic placement, GCF novelty, synteny conservation, mobilome context, and self-resistance/transporter linkages, genome-guided discovery in *Lentzea* spp. can be directed toward an evolution-informed, targeted search for novel natural products.

## 4. Materials and Methods

### 4.1. Genome Data Collection, Sequencing, Taxonomic Assignment, and Quality Control

The overall quality control workflow is summarized in Figure S1. The starting dataset used to select *Lentzea* genomes was compiled from two sources: publicly available genomes from NCBI RefSeq and self-sequenced isolates generated in this study. As of 1 October 2025, 874 genomes assigned to the family *Pseudonocardiaceae* were downloaded from NCBI RefSeq (Table S2). Metagenome-assembled genomes were excluded during database retrieval using the NCBI Genome filtering options.

For the self-sequenced isolates (7 strains), genomic DNA was extracted using a modified cetyltrimethylammonium bromide method, and DNA quantity, purity, and integrity were evaluated before sequencing. Whole-genome sequencing and genome assembly were performed by Personal Biotechnology (Shanghai, China) using a hybrid short-read and long-read strategy. The final complete circular genome sequences and their assembly quality metrics were incorporated into the genome dataset for subsequent comparative analyses.

Genome completeness and contamination were estimated with CheckM [49]. Genomes with completeness ≤90% or contamination ≥5% were classified as low quality and removed from downstream analysis. This filtering step removed 32 genomes from 874 candidate genomes, leaving 842 high-quality genomes. In the same process, the genomes of 7 self-sequenced isolates were assessed with CheckM (Figure S1A). For two of the seven isolates we sequenced (GCF_014653695.1 and GCF_014646255.1), the RefSeq draft genomes were also substituted with our own assemblies.

To reduce redundancy in the public dataset before downstream analysis, genomes explicitly annotated as the same strain in the accompanying metadata were dereplicated via exact strain name matching. When multiple assemblies were available for the same strain, the representative genome was selected according to the following priority: higher assembly level, higher N50 within the same assembly level, and more recent submission date. A total of 809 genomes were retained as *Pseudonocardiaceae* (Figure S1D).

All retained genomes were re-annotated with Prokka [44] to standardize gene prediction, locus tags, and downstream coordinate systems.

All compiled genomes from NCBI RefSeq and the self-sequenced isolates were classified using GTDB-Tk [50] against the Genome Taxonomy Database [51]. Genomes not assigned to the family *Pseudonocardiaceae* by GTDB-Tk were excluded. Within the retained family-level dataset, genomes classified as the genus *Lentzea* were defined as the genus-focused dataset. After GTDB-Tk classification, 808 genomes were retained as *Pseudonocardiaceae*, including 64 genomes assigned to *Lentzea* spp. (Figure S1B).

Because metadata-based strain matching cannot detect all repeatedly sequenced or incompletely registered clonal isolates, the background dataset was further dereplicated with dRep [52] using FastANI-based clustering at 99.5% average nucleotide identity [53]. Representative genomes were selected using dRep quality scoring with additional consideration of assembly continuity. After quality control and dereplication, the final non-redundant family-level dataset comprised 652 high-quality *Pseudonocardiaceae* genomes, including 59 *Lentzea* genomes used for genus-focused analyses.

### 4.2. Genome Datasets and Outgroup Selection

The final family-level dataset consisted of 652 high-quality, non-redundant *Pseudonocardiaceae* genomes, whereas the genus-level analyses were based on 59 quality-controlled *Lentzea* genomes. Taxonomic assignments throughout the study followed GTDB-Tk results. The framework of Teo et al. (2021) [54] was used to guide family-level phylogenomic context and outgroup selection, not to replace GTDB-based taxonomic classification. For family-level phylogenomics, three *Corynebacteriales* genomes, *Gordonia bronchialis* DSM 43247, *Tsukamurella paurometabola* DSM 20162, and *Nocardia asteroides* NCTC 10356, were used as outgroups. *Saccharothrix espanaensis* DSM 44229 was used as the outgroup for *Lentzea*-focused phylogenomics.

### 4.3. Family-Level Phylogenomic Analysis

A genome-wide phylogeny of the family *Pseudonocardiaceae* was inferred using GToTree [55] with an actinobacteria-specific marker gene set. Briefly, single-copy marker proteins were identified (by HMMER3) [56], aligned (by Muscle) [57], automatically trimmed (by trimAl) [58], and concatenated into a supermatrix. Genomes with <50% marker gene coverage were excluded, resulting in an alignment of 655 genomes (652 *Pseudonocardiaceae* genomes and three outgroups) for phylogenetic inference. A maximum likelihood tree was constructed with FastTree [59] and rooted using the three *Corynebacteriales* outgroups. Genus-level taxonomy, genome size, and CDS counts were mapped onto the tree to summarize taxonomic placement and genome features across the family.

### 4.4. Lentzea Phylogenomics and Robustness Assessment

To resolve relationships among *Lentzea* spp. and its close relatives, orthology-based phylogenomics was performed using OrthoFinder [60] on 59 *Lentzea* genomes and *Saccharothrix espanaensis* DSM 44229. The OrthoFinder workflow incorporated DIAMOND [61] for protein similarity searches and MAFFT [62] for multiple-sequence alignment. Single-copy orthologous proteins shared by all taxa were extracted, aligned, trimmed, and concatenated into a final supermatrix of 1719 aligned single-copy orthologous groups (amino acid positions). Maximum-likelihood inference was conducted with IQ-TREE [63] under the best-fit model selected by ModelFinder [64], with branch support assessed via ultrafast bootstrap [65]. To assess sensitivity to model selection, an additional maximum-likelihood analysis was performed under the LG+F+R9 model and compared with the best-model topology (Figure S3). The final *Lentzea* tree was rooted using *S. espanaensis* DSM 44229.

### 4.5. Whole-Genome ANI and Phylogeny ANI Congruence

Pairwise whole-genome average nucleotide identity among the 59 *Lentzea* genomes was calculated using the ANIb method in pyANI [66]. The ANI matrix was converted into a distance matrix using d = 1 − ANI, and an UPGMA hierarchical clustering tree was generated. To compare genome-wide similarity patterns with core genome phylogeny, the IQ-TREE phylogeny was converted to an ultrametric tree while preserving topology, and a tanglegram was used to visualize concordance or conflict between the two tree structures.

### 4.6. Pangenome Reconstruction and Profiling

The *Lentzea* pangenome was first reconstructed using IPGA [33], which integrates multiple pangenome engines and evaluates candidate orthogroup partitions using internal consistency scoring. Pangenome and core genome accumulation curves were generated by permutation-based sampling, and pangenome openness was estimated by fitting Heaps’ law to the pangenome curve and an exponential decay model to the core genome curve. The IPGA-selected OrthoFinder profile was used to generate the pangenome and core genome accumulation curves. Orthogroups were classified by prevalence across the 59 *Lentzea* genomes as core (>95%; equivalent to presence in at least 57/59 genomes), shell (15–95%), or cloud (<15%); shell and cloud orthogroups were collectively considered the accessory genome (Table S4).

### 4.7. Functional Annotation and COG-Based Comparisons

Representative protein sequences from each orthogroup were functionally annotated with eggNOG-mapper [67] against the eggNOG database [68]. COG category distributions were summarized separately for the core and accessory genomes, with emphasis on functional categories related to secondary metabolism, transcriptional regulation, and environmental adaptation (Table S5).

### 4.8. Identification of Candidate Lentzea-Specific Strict Core Genes

Candidate *Lentzea*-specific strict core genes were identified using a two-step filtering strategy. First, strict core orthogroups present in all 59 *Lentzea* genomes were retained. Second, the corresponding protein sequences were queried against a non-*Lentzea* background database comprising 744 *Pseudonocardiaceae* genomes from other genera (the pre-dRep set of 808 genomes, including 64 *Lentzea* and 744 non-*Lentzea* genomes, distinct from the dRep-deduplicated dataset of 652 genomes, including 59 *Lentzea* and 593 non-*Lentzea* genomes, used elsewhere). This non-deduplicated collection ensured the widest range of genes for comparison. Hits satisfying identity ≥ 30%, coverage ≥ 50% (query coverage), and E-value ≤ 1 × 10^−5^ were considered homologous and removed. Orthogroups retained after both filters were defined as candidate *Lentzea*-specific strict core genes and summarized by functional category.

### 4.9. Biosynthetic Gene Cluster Prediction and Comparative Statistics

Secondary metabolite biosynthetic gene clusters (BGCs) were predicted for all genomes using antiSMASH [2]. Because all genomes had been standardized with Prokka, antiSMASH was configured to use existing gene coordinates from the standardized GenBank files. Similarity to characterized BGCs was evaluated against MIBiG [8] through the ClusterBlast and known-cluster comparison modules. AntiSMASH product labels were harmonized into six broad BGC categories: Terpene, NRPS, PKS, RiPPs, Saccharide, and Other (Table S6). The analysis procedure of the BGCs integrated the GNU Parallel, data parsing and statistical analysis to ensure efficient and accurate handling of the large-scale datasets.

To compare BGC profiles between *Lentzea* spp. and the remaining family members, genomes were grouped as *Lentzea* (*n* = 59) and non-*Lentzea Pseudonocardiaceae* genomes (*n* = 593). BGC abundance per genome was compared using the Wilcoxon rank-sum test. BGC composition was summarized as both mean counts and relative proportions of standardized BGC categories. Associations between genome size and total BGC number were evaluated using correlation analysis and linear regression.

### 4.10. Gene Cluster Family Clustering, Novelty Assessment, and Network Visualization

To evaluate BGC diversity across the family, all predicted BGCs from the 652 *Pseudonocardiaceae* genomes were clustered together with 2632 reference BGCs from MIBiG using BiG-SCAPE [6] in mixed mode.

Here, a BGC denotes an individual predicted biosynthetic region; a GCF denotes a similarity-based family assignment produced by BiG-SCAPE; a CC denotes a connected component of the BGC similarity network; and an OG denotes an OrthoFinder orthogroup used for gene-level conservation and architecture analyses. GCF and CC assignments were retained as distinct analytical units.

To select a single BiG-SCAPE distance cutoff for the principal analyses, the BGCs were clustered independently with BiG-SCAPE v2.0.0 at distance cutoffs of 0.10, 0.15, 0.20, 0.25 and 0.30, while retaining the same input records and other analysis settings. MIBiG reference records were excluded from the taxonomic and component size summaries. For each cutoff, we quantified the numbers of gene-cluster families (GCFs) and connected components (CCs), the fraction of singleton GCFs, the fractions of BGCs assigned to mixed-genus GCFs and CCs, and the size and taxonomic breadth of the largest GCF and CC. For *Lentzea*, we additionally calculated the fractions of BGCs assigned to genus-specific GCFs and taxonomically single-genus CCs. A genus-specific GCF was defined as a GCF, whose non-MIBiG members were restricted to the genus of the focal BGC, whereas a single-genus CC contained BGCs from only one genus. Network layouts at all five cutoffs were also inspected for fragmentation and the formation of disproportionately large multi-genus components.

Cutoff 0.20 was retained as the principal setting because it provided an empirical balance between the extensive fragmentation observed at 0.10–0.15 and the progressive merging of distinct, taxonomically broad components observed at 0.25–0.30 (Figure S4 and Table S11). As a further sensitivity analysis relevant to Result 5, the six candidate CCs defined at cutoff 0.20 were traced at cutoffs 0.25 and 0.30 by shared BGC membership. For each anchor candidate, the CC containing the largest number of its original BGCs was identified, and its target genus fraction, number of non-target genera and co-occurrence with other cutoff-0.20 anchor candidates were recorded. A likely cutoff-dependent overmerge was flagged when a family-scale CC contained more than one cutoff-0.20 anchor candidate and members from more than ten non-target genera. Cutoffs 0.25 and 0.30 were therefore used only for sensitivity assessment and discovery-oriented inspection, whereas all principal GCF/CC definitions and downstream analyses used cutoff 0.20.

GCF and CC assignments at a distance cutoff of 0.20 were retained separately, and sequence similarity networks were visualized in Cytoscape v3.10.3 [69]. A group was considered genus-specific when its non-MIBiG BGC members originated from a single genus, assessed separately for GCFs and CCs. Within-genus prevalence was calculated as the proportion of genomes carrying at least one BGC assigned to the relevant GCF or CC. Four prevalence tiers were used: genus-conserved (≥90%), broadly shared (≥50% to <90%), narrowly distributed (≥5% to <50%), and strain-restricted (<5%). GCF-based tiers from the *Lentzea*-focused clustering were used for the MGE association analyses in Figure 5B and Tables S17–S19. CC-based tiers and single-genus CC membership were used for the genus-specific repertoire summaries in Figure 5C, Figure S6, and Table S12.

To estimate biosynthetic novelty while reducing the effect of uneven genome sampling among genera, genus-level comparisons used average BGC counts per genome rather than raw group counts. BGCs lacking MIBiG-connected GCF or CC assignments were classified as putatively novel according to Table S8. Within the single-genus CC subset defined in Table S12, putatively novel BGCs were summarized by genus and BGC category as the mean number per genome and the mean BGC length (Figure 5D; Table S13). Only genera represented by >7 genomes, corresponding to >1% of the 652-genome dataset, were included in inter-genus comparisons (Tables S6 and S9).

### 4.11. OG-Informed Selection and Domain Architecture Analysis of Lineage-Associated BGC Candidates

OG assignments from the standalone OrthoFinder analysis were used for BGC-associated gene mapping and the conservation and domain architecture analyses described below, separately from the IPGA-selected profile used for accumulation curve analysis.

BiG-SCAPE c0.20 CCs from the 59-genome *Lentzea* dataset were linked to OrthoFinder OGs using standardized gene identifiers and BGC coordinates. Candidate CCs were required to meet the following criteria: occur in at least 50% of the genomes; have a core biosynthetic gene OG mapping fraction of at least 0.90; contain at least one BGC-embedded core OG recurring in at least 90% of CC member strains; have consensus core OG completeness of at least 0.90, median pairwise core OG-set Jaccard similarity of at least 0.75, and a consensus exact single copy fraction of at least 0.80; contain at least 80% non-edge BGC regions; and have detected mobile genetic element (MGE) evidence in no more than 20% of member BGCs. Genus- and family-scale CCs were required to lack linked MIBiG records. The corresponding CC in the 652-genome family dataset was also required to contain no non-*Lentzea* BGC members. CCs with a prevalence of at least 0.80 were classified as genus-wide. CCs with a prevalence between 0.50 and 0.79 were retained as clade-associated candidates when their species tree presence/absence pattern had permutation support (*p* ≤ 0.05). OG recurrence and CC prevalence defined the primary conservation axis. Completeness and MGE criteria were used for quality control and secondary prioritization. CCs meeting all selection criteria were prioritized using the conservation and quality metrics summarized in Data S4, Table S14.

For gene content analysis, BGC regions touching an antiSMASH record boundary were excluded from variable OG denominators. OGs were scored as present or absent in each evaluable BGC. Selected regulatory and tailoring/additional biosynthetic OGs were displayed alongside the species tree. Representative BGCs were rigidly reversed when necessary and translated to a common 0 kb origin while preserving alignment of the domain-variable OG (Figure 6B; Supplementary Figure S8; Data S4, Table S15).

Pfam architectures were reconstructed from accession-specific InterProScan [70] and Pfam hits ordered by protein coordinates. Recurrent events met the recurrence and sequence quality criteria listed in Data S4, Table S16. Associations between architecture state and the six host clades were tested using a Monte Carlo Fisher exact test with 10,000 replicates, with Cramér’s V reported as the effect size. Phylogenetic clustering was assessed by calculating the minimum multistate parsimony steps on the 59-genome species tree and comparing the observed value with 2000 random permutations of architecture labels among the observed strains. The lower-tail permutation *p* value was calculated as (1 + the number of null values less than or equal to the observed number of steps)/(1 + the number of valid permutations). Fisher and phylogenetic *p* values were adjusted across tested events using the Benjamini–Hochberg method. These analyses quantified clade association and phylogenetic clustering of the observed architecture states (Data S4, Table S16).

### 4.12. Identification and Integration of Mobilome-Associated BGCs

To investigate the potential contribution of mobile genetic elements (MGEs) to secondary metabolite diversification in *Lentzea* spp., an integrative genome-mining framework was used to identify BGCs associated with genomic mobility signatures. Genomic islands were predicted with IslandViewer4 [45], whereas plasmid- and virus/phage-associated regions were identified with geNomad [46]. Mobile element-related genes, including transposases, integrases, and recombinases, were detected from Prokka annotations and antiSMASH SMCOG annotations within BGCs and their 10 kb flanking regions. Spatial overlap analysis was then performed between predicted MGE coordinates and antiSMASH-defined BGC boundaries.

To reduce false positives from any single prediction source, a binary evidence matrix was constructed using five mobility-related criteria: genomic island evidence within BGCs, Prokka-annotated mobile genes within or adjacent to BGCs, antiSMASH mobile-related SMCOGs, overlap with geNomad-predicted plasmid regions, and overlap with geNomad-predicted phage or viral regions. BGCs matching at least one criterion were classified as putatively MGE-associated, whereas those matching none of the five criteria were classified as BGCs without detected MGE context evidence. These categories were further stratified by BiG-SCAPE-derived GCF prevalence tier and MIBiG similarity to evaluate the relationship among mobility, prevalence, and chemical novelty (Figure S9, Table S17).

### 4.13. Mobilome-Associated BGC Classification

Putatively MGE-associated BGCs were identified using five evidence types: overlap with predicted genomic islands, Prokka-annotated mobile element genes within or near BGCs, antiSMASH mobile-related SMCOGs, overlap with geNomad-predicted plasmid regions and overlap with geNomad-predicted phage or viral regions. BGCs supported by at least one evidence type were classified as putatively MGE-associated, whereas those without support from any of the five evidence types were classified as BGCs without detected MGE context evidence. A stricter threshold requiring at least two evidence types was used as a sensitivity criterion. MGE-associated fractions were summarized by BiG-SCAPE GCF prevalence tier and evaluated with Fisher’s exact tests and a Cochran–Armitage trend test (Table S18). For Table S18, Benjamini–Hochberg correction was applied jointly to the two Fisher exact tests, the Cochran–Armitage trend test, and the Kruskal–Wallis test, and separately to six clade-versus-rest Wilcoxon comparisons. Adjusted *p*-values are reported as q-values, with q < 0.05 used for interpretation.

### 4.14. Screening of BGC-Associated Resistance Mechanisms

To identify candidate self-resistance mechanisms associated with secondary metabolite biosynthesis, a hierarchical screening pipeline integrating ARTS [71], CARD-RGI [72], and TCDB [73] was applied to the proteomes of 59 *Lentzea* genomes. ARTS was used to detect duplicated or divergent core housekeeping genes potentially involved in target-directed resistance. CARD-RGI was used to identify classical resistance and modification mechanisms, retaining high-confidence Strict or Perfect hits. To capture transporter-mediated defense mechanisms that may be underrepresented in clinical resistance databases, a customized BLASTp search was also performed against TCDB, followed by filtering for multidrug, efflux, toxin, antibiotic, and heavy metal transport-related families.

To distinguish putative BGC-associated self-resistance genes from background resistance determinants, all candidates detected by ARTS, CARD-RGI, and TCDB were mapped to antiSMASH-defined BGC coordinates. Genes located within BGC regions or within 10 kb upstream or downstream flanking regions were considered spatially coupled to BGCs. Overlap among the three resistance detection approaches was quantified and visualized to evaluate their complementarity (Table S20).

### 4.15. Phylogenomic Mapping of Mobilome-Associated BGCs

To assess lineage-specific patterns of mobility-associated secondary metabolism, the abundance and biosynthetic classes of putatively MGE-associated BGCs were mapped onto the *Lentzea* maximum likelihood phylogeny. For each genome, MGE-associated BGCs were summarized by major biosynthetic class, including RiPPs, terpenes, NRPS, PKS, and other categories, and visualized alongside the phylogenomic tree as aligned bar plots or heatmaps. Major phylogenomic lineages (Clades I–VI) were annotated to identify clade-level enrichment of mobility-associated BGCs and intra-genus variation in genomic plasticity.

### 4.16. Statistical Analysis and Visualization

Unless otherwise specified, downstream parsing, statistical analysis, and figure generation were performed in R. General plots were generated with ggplot2, tree annotations with ggtree-related packages and iTOL where appropriate, tanglegrams with dendextend or phytools, and set overlap visualizations with UpSet-based tools. Comparisons of biosynthetic gene cluster architectures were visualized using clinker [74]. Multi-panel figures were assembled using R-based plotting utilities. Network layouts were generated in Cytoscape v3.10.3. Software versions are listed in Table S1.

## 5. Conclusions

In summary, our family-scale comparative genomic analysis and genus-focused characterization of the pangenome, BGCs, and mobilome reveal that the rare actinomycete genus *Lentzea* harbors a dense, taxonomically distinctive, and largely underexplored specialized metabolic repertoire. *Lentzea* spp. exhibit an open pangenome with numerous putatively novel and genus-associated GCFs. By integrating lineage-associated BGC conservation with mobility-associated genomic contexts, we propose a dual-pattern model of BGC conservation and mobility-associated diversification. In this model, conserved lineage-associated BGC groups represent candidates for genus signature chemistry, whereas the enrichment of MGE-associated contexts among strain-restricted and narrowly distributed GCFs highlights clade- and strain-level biosynthetic diversity. More broadly, by integrating phylogenomics, pangenomics, GCF novelty, synteny conservation, and mobilome context, this study provides a targeted comparative genomic foundation for natural product discovery in rare actinomycetes.

## Data Availability

The newly generated genome assemblies have been deposited in NCBI BioProject under PRJNA1496027. Public genome assemblies analyzed in this study are identified by accession in Additional file 2 (Data S1; Table S2). Derived data supporting the figures, statistical analyses and main conclusions are provided in Additional files 1–6 and in zenodo at 10.5281/zenodo.21771150. Source code and analysis workflows corresponding to this article are also available at 10.5281/zenodo.21771150. The private link and access instructions: https://zenodo.org/records/21771150 (accessed on 27 September 2026).

## Supplementary Materials

The following supporting information can be downloaded at: https://www.mdpi.com/article/10.3390/antibiotics15100967/s1. The supporting information consists of a supplementary document containing Figures S1–S11 and Table S1, together with five machine-readable workbooks. Data S1 contains genome metadata, quality control, dataset membership and curated habitat evidence (Table S2). Data S2 contains pangenome composition and functional analysis (Tables S3–S5). Data S3 contains family-scale BGC profiling, novelty, and genus specificity (Tables S6–S13). Data S4 contains conservation and domain architecture analyses of lineage-associated BGC candidates (Tables S14–S16). Data S5 contains MGE context and resistance/transporter analyses (Tables S17–S20).

## Abbreviations

The following abbreviations are used in this manuscript:

BGC	Biosynthetic gene cluster
CC	Connected component
GCF	Gene cluster family
MGE	Mobile genetic element
NRPS	Nonribosomal peptide synthetase
OG	Orthogroup
PKS	Polyketide synthase
RiPP	Ribosomally synthesized and post-translationally modified peptide
ANI	Average nucleotide identity
ANIb	BLAST-based average nucleotide identity
COGs	Clusters of Orthologous Groups
CDS	Coding sequence
MIBiG	Minimum Information about a Biosynthetic Gene cluster
SMCOG	Secondary metabolite clusters of orthologous groups
TCDB	Transporter Classification Database
CARD	Comprehensive Antibiotic Resistance Database
ARTS	Antibiotic-Resistant Target Seeker
GTDB	Genome Taxonomy Database
