# Supplementary material for: Deciphering the Genome Evolution and Cryptic Biosynthetic Potential in Rare Lentzea spp
Source: Antibiotics (Basel). 2026 Sep 30;15(10):967. doi: 10.3390/antibiotics15100967 (PMC13700613; doi:10.3390/antibiotics15100967)
Supplement: Supplementary file 1 [file antibiotics-15-00967-s001.zip › Supplementary Figures.pdf]

Supplementary Information

Table of Contents

Table S1. Software and databases used in this study. 2

Figure S1. Dataset construction, taxonomic reassignment, and genome-quality overview. 4

Figure S2. Phylogenomic relationships and ecological-source annotations of the curated *Lentzea* genomes. 5

Figure S3. Robustness of *Lentzea* phylogenomic topology under Q.INSECT and LG substitution models. 6

Figure S4. Selection of the BiG-SCAPE cutoff used in the principal analyses. 7

Figure S5. Family-wide BiG-SCAPE network of 652 dereplicated *Pseudonocardiaceae* genomes at cutoff 0.20. 8

Figure S6. Mobility context of CC-level *Lentzea*-specific BGCs across conservation tiers. 9

Figure S7. Complete intrageneric distribution of six potential-vertical candidate connected components. 10

Figure S8. Complete gene-content and domain-architecture variation in CC00231 and CC00158. 11

Figure S9. Intersection structure of five evidence dimensions used to classify MGE-associated BGCs. 12

Figure S10. Intersection and class-level distribution of BGC-linked resistance evidence. 13

Figure S11. Mobility-associated flow of the *Lentzea* secondary metabolome. 14

Data S1. Genome metadata, quality control and dataset membership. 15

Data S2. Pangenome composition and functional analysis. 15

Data S3. Family-scale BGC profiling, novelty and genus specificity. 15

Data S4. Potential-vertical candidate BGC conservation and domain architecture. 15

Data S5. MGE-associated and resistance-linked BGC analyses. 15

References 16

Table S1. Software and databases used in this study.

No.	Tool name	Version	Description	Link	Refs
1	CheckM	1.2.4	Assess genome completeness and contamination.	https://github.com/Ecogenomics/CheckM	[1]
2	GTDB-Tk	2.6.1	Assign genome taxonomy using the GTDB reference framework.	https://github.com/Ecogenomics/GTDBTk	[2,3]
3	dRep	3.4.2	Dereplicate genomes using quality metrics and pairwise ANI.	https://github.com/MrOlm/drep	[4]
4	fastANI	1.34	Estimate whole-genome average nucleotide identity rapidly.	https://github.com/ParBLiSS/FastANI	[5]
5	Prokka	1.14.6	Annotate bacterial genomes and generate standardized GenBank/GFF/protein files.	https://github.com/tseemann/prokka	[6]
6	eggNOG-mapper	2.1.13	Assign orthology-based COG, KEGG, GO, CAZy and Pfam functional annotations.	https://github.com/eggnogdb/eggnog-mapper	[7,8]
7	OrthoFinder	3.1.0	Infer orthogroups, single-copy orthologues and species-tree inputs.	https://github.com/davidemms/OrthoFinder	[9]
8	DIAMOND	2.1.17	Perform fast protein similarity searches.	https://github.com/bbuchfink/diamond	[10]
9	MAFFT	7.526	Align protein sequences for orthology and phylogenomic workflows.	https://mafft.cbrc.jp/alignment/software/	[11]
10	FastTree	2.1.11	Infer approximate maximum-likelihood trees from sequence alignments.	http://www.microbesonline.org/fasttree/	[12]
11	GToTree	1.8.16	Make large-scale phylogenomic tree	https://github.com/AstrobioMike/GToTree	[13]
12	IQ-TREE	3.0.1	Infer maximum-likelihood phylogenies with model selection and ultrafast bootstrap support.	https://iqtree.github.io/	[14]
13	pyani	0.2.13v1	Calculate ANIb matrices for clone-level genome comparison.	https://github.com/widdowquinn/pyani	[15]
14	antiSMASH	8.0.0	Predict and annotate biosynthetic gene clusters and known-cluster similarity.	https://antismash.secondarymetabolites.org/	[16]
15	GNU parallel	20251122	Run antiSMASH jobs in parallel with resumable job logging.	https://www.gnu.org/software/parallel/	
16	BiG-SCAPE	2.0.0	Cluster BGCs into gene-cluster families	https://github.com/	[17,18]

No.	Tool name	Version	Description	Link	Refs
			and connected components across multiple cutoffs.	medema-group/BiG-SCAPE	
17	MIBiG database	4.0	Provide curated reference biosynthetic gene clusters for novelty-context assessment.	https://mibig.secondarymetabolites.org/	[19]
18	clinker	0.0.32	Visualize gene-cluster synteny and export pairwise gene-alignment links.	https://github.com/gamcil/clinker	[20]
19	InterProScan	5.77-108.0	Annotate protein domains, signatures, InterPro entries and GO terms.	https://www.ebi.ac.uk/interpro/about/interproscan/	[21]
20	IslandViewer 4	As of 2026.03	Predict genomic islands and provide interval/gene calls.	https://www.pathogenomics.sfu.ca/islandviewer/	[22]
21	geNomad	1.11.2	Identify plasmid, virus and provirus sequences in genomic assemblies.	https://github.com/apcamargo/genomad	[23]
22	ARTS	3.0b2	Predict candidate antibiotic-resistance targets associated with BGCs.	https://github.com/ZiemertLab/ARTS	[24]
23	Resistance Gene Identifier	6.0.7	Detect antimicrobial-resistance genes against the CARD reference database.	https://github.com/arpcard/rgi	[25]
24	CARD database	4.0.1	Provide curated antimicrobial-resistance ontology and reference sequences.	https://card.mcmaster.ca/	[25]
25	TCDB	As of 2026.03	Provide transporter-family references for export, efflux and resistance-context annotation.	https://www.tcdb.org/	[26]
26	IPGA	1.09	Provide external prokaryotic genome/pangenome analyses and network or tree exports.	https://nmdc.cn/ipga/	[27]
27	Cytoscape	3.10.3	Visualize and manually refine large BGC similarity networks.	https://cytoscape.org/	[28]

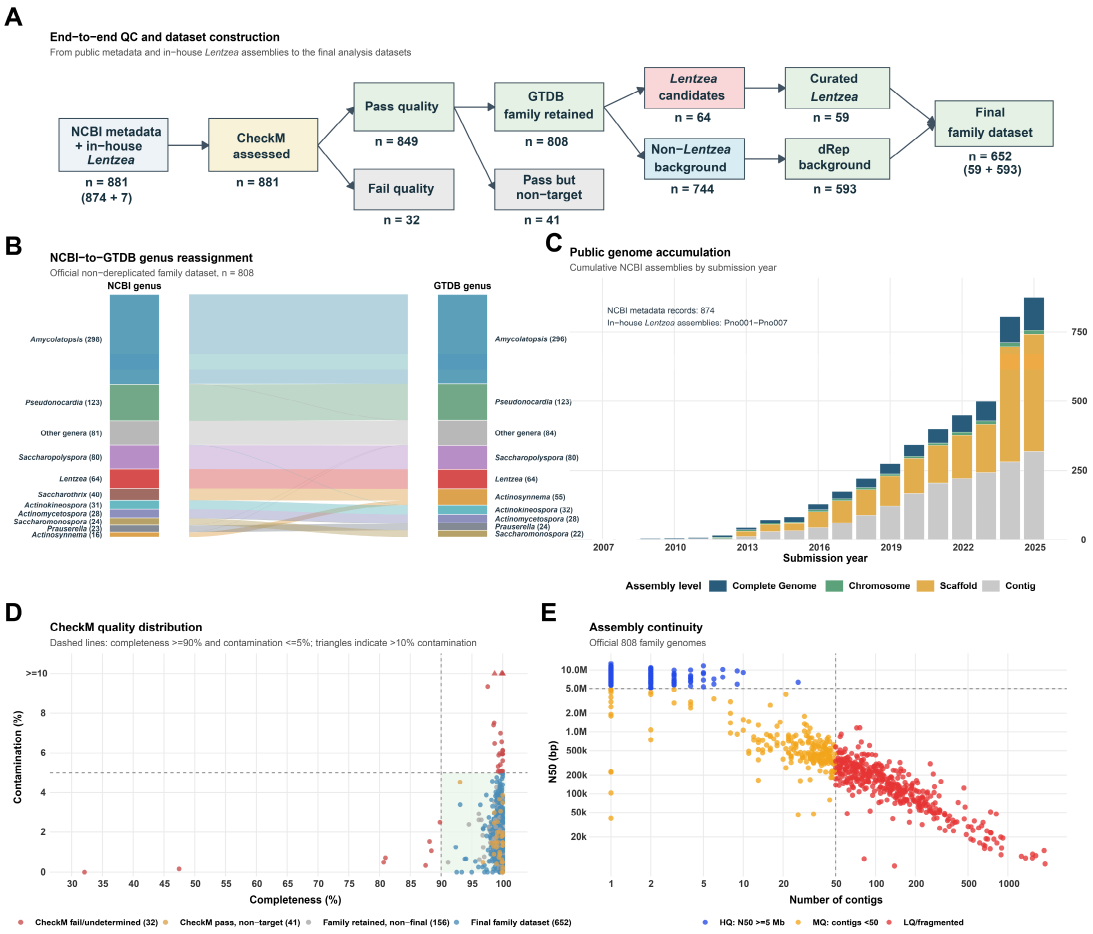

Figure S1. Dataset construction, taxonomic reassignment, and genome-quality overview.

The workflow summarizes genome collection, quality control, GTDB-based family retention, *Lentzea* curation, and dereplication steps used to define the final family-scale and genus-scale datasets.

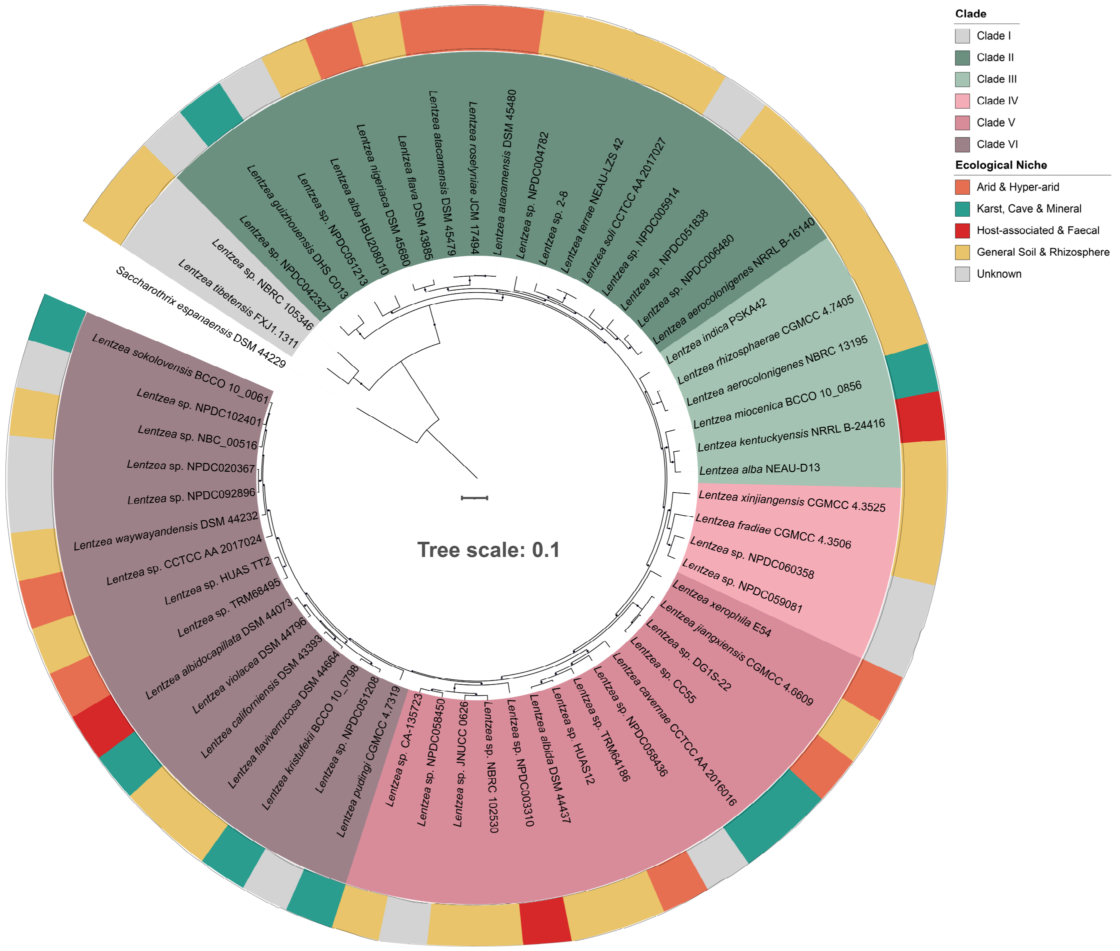

Figure S2. Phylogenomic relationships and ecological-source annotations of the curated *Lentzea* genomes.

The circular phylogeny shows six *Lentzea* clades, with the outer ring indicating available ecological-source categories (arid/hyper-arid, karst/cave/mineral, host-associated/faecal, general soil/rhizosphere, or unknown).

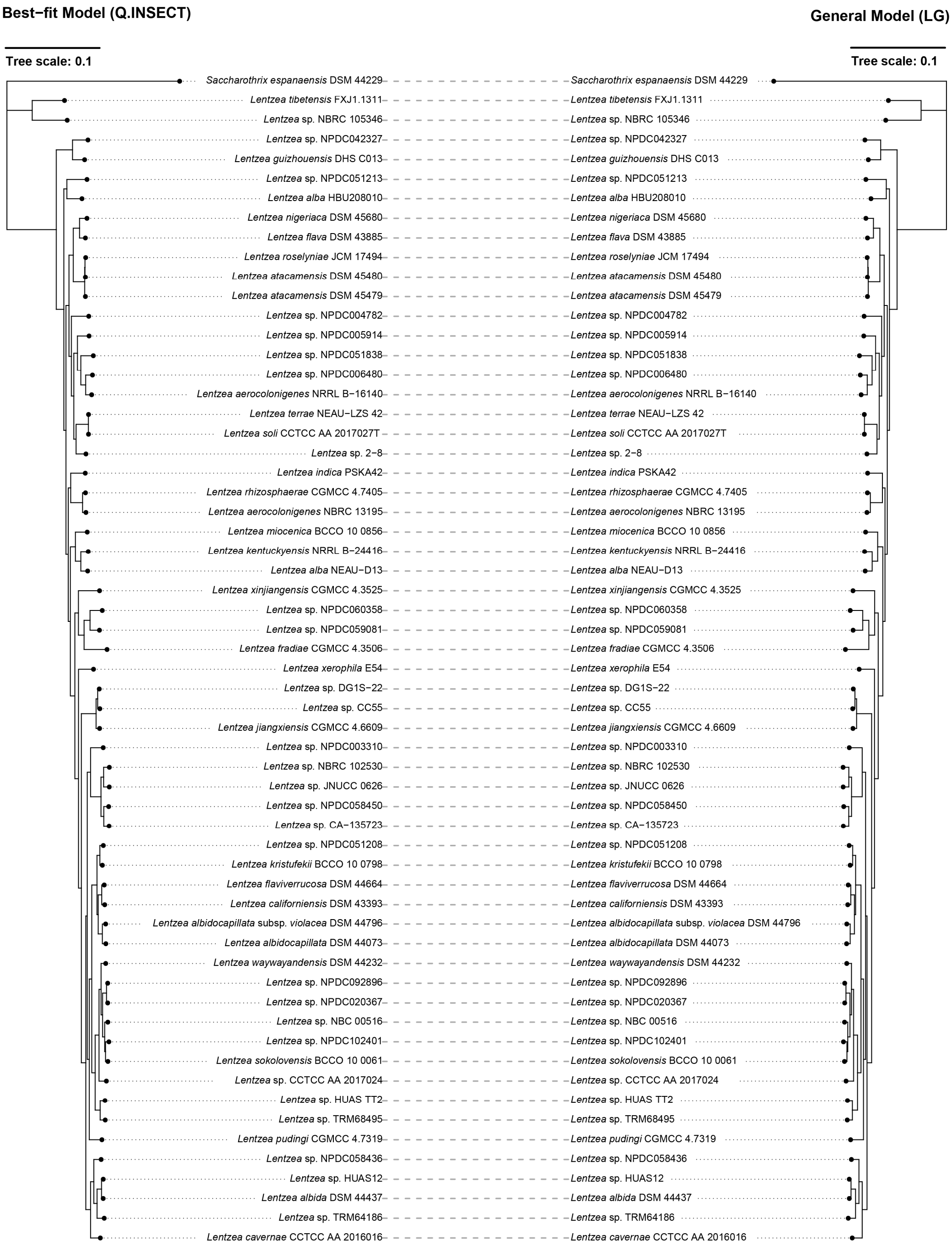

Figure S3. Robustness of *Lentzea* phylogenomic topology under Q.INSECT and LG substitution models.

The tanglegram compares the best-fit Q.INSECT model with the general LG model for the curated *Lentzea* dataset. Dashed links connect identical genomes; broadly concordant branching supports the stability of the major genus-level phylogenomic framework across model choices.

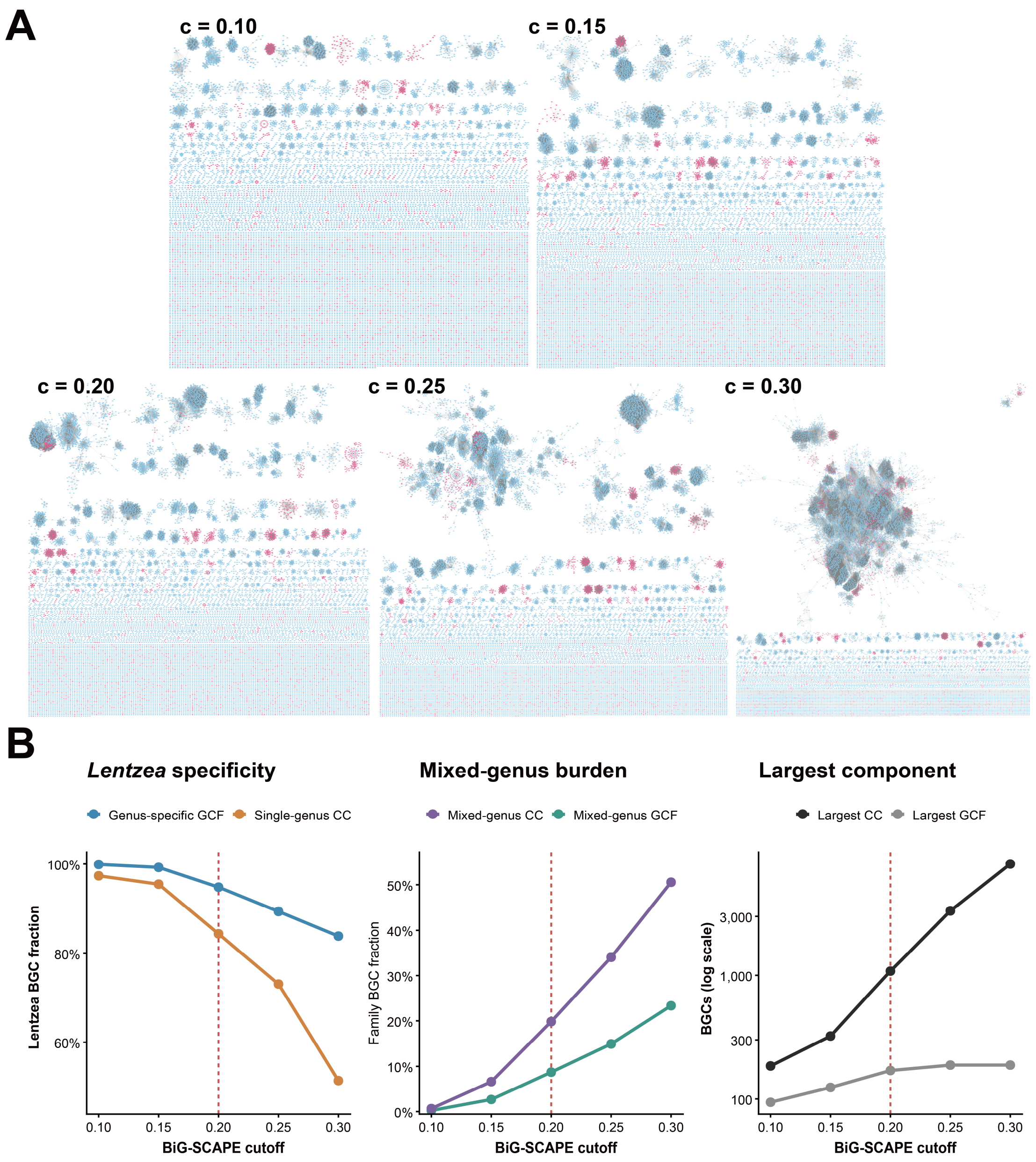

Figure S4. Selection of the BiG-SCAPE cutoff used in the principal analyses.

(A) Family-scale BiG-SCAPE networks at cutoffs 0.10, 0.15, 0.20, 0.25, and 0.30. Target-genus BGCs are highlighted in red. (B) Quantitative sensitivity analysis of *Lentzea* specificity, mixed-genus component burden, and the largest family-scale component. At cutoff 0.20, 94.8% of *Lentzea* BGCs were assigned to genus-specific GCFs, 84.4% occurred in single-genus connected components, 19.8% of family BGCs occurred in mixed-genus connected components, and the largest connected component contained 1,083 BGCs. More permissive cutoffs increased mixed-genus burden and expanded the largest connected component to 3,328 BGCs at 0.25 and 7,946 BGCs at 0.30. Cutoff 0.20 was therefore retained as a reproducible compromise between fragmentation at stricter thresholds and over-merging at more permissive threshold.

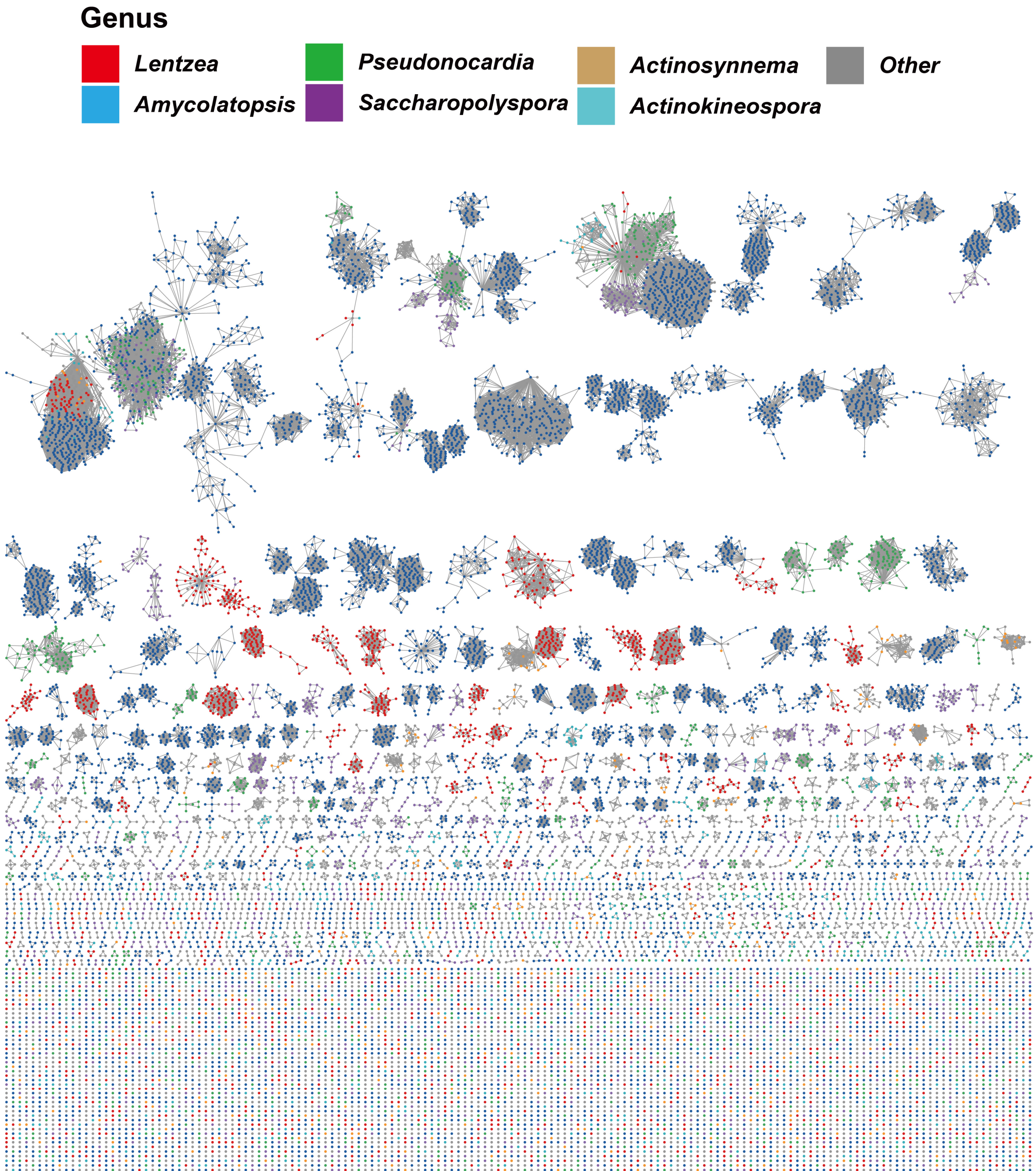

Figure S5. Family-wide BiG-SCAPE network of 652 dereplicated *Pseudonocardiaceae* genomes at cutoff 0.20.

Nodes represent BGC regions and edges represent pairwise similarity relationships passing the selected cutoff; node colours denote source genus.

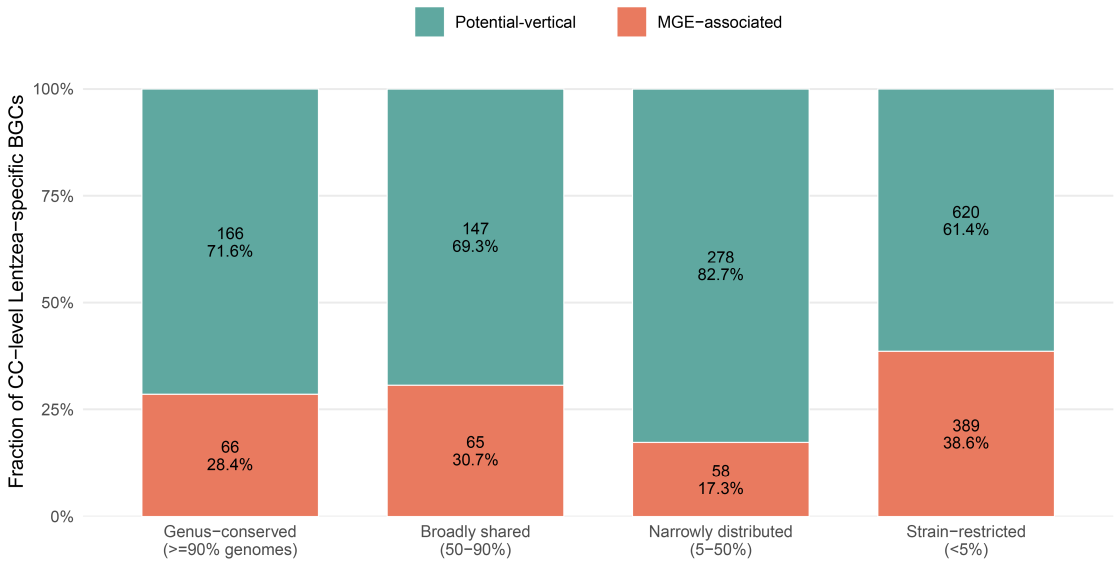

Figure S6. Mobility context of CC-level *Lentzea*-specific BGCs across conservation tiers.

Stacked bars show the numbers and fractions of potential-vertical and MGE-associated BGCs within the CC-level *Lentzea*-specific subset at BiG-SCAPE cutoff 0.20. *Lentzea*-specific BGCs were defined at the connected-component level rather than the GCF level to avoid overestimating genus specificity under stringent clustering. MGE-associated BGCs were detected across all four conservation tiers and were most frequent in the Strain-restricted tier.

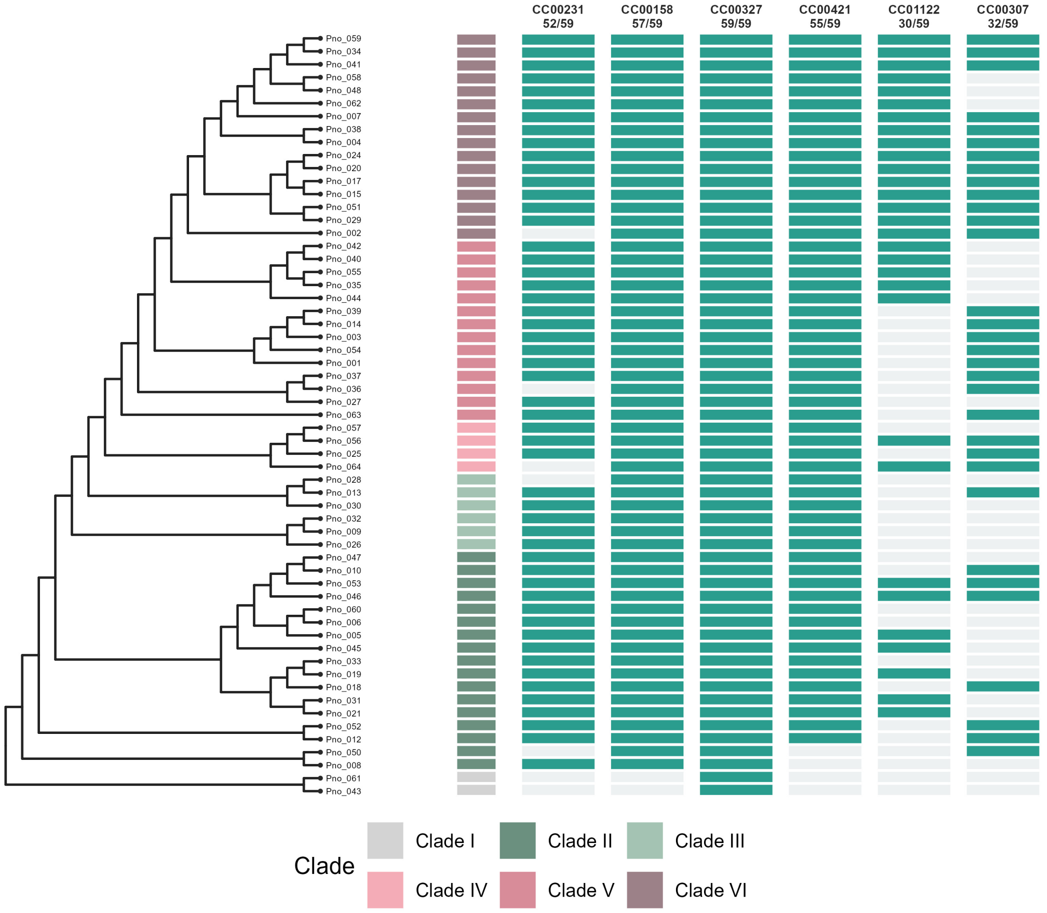

Figure S7. Complete intrageneric distribution of six potential-vertical candidate connected components.

Presence or absence of a BGC from CC00231, CC00158, CC00327, CC00421, CC01122, and CC00307 is mapped onto the 59-genome *Lentzea* species tree. Candidate order follows Fig. 5A, and column labels indicate the number of genomes carrying each CC. Teal denotes presence, and pale grey denotes absence.

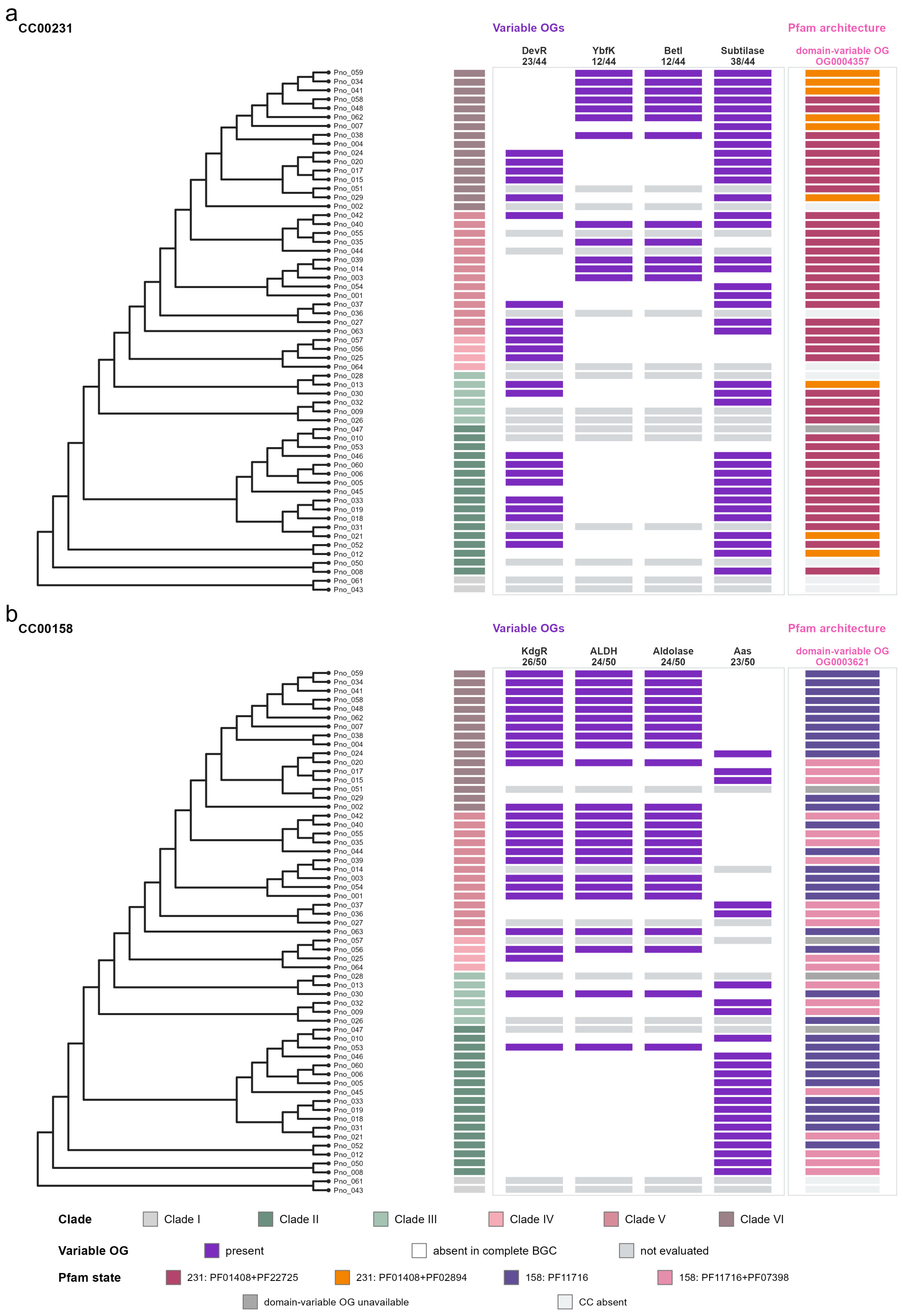

Figure S8. Complete gene-content and domain-architecture variation in CC00231 and CC00158.

(A) CC00231. (B) CC00158. Each panel aligns the 59-genome Lentzea species tree and clade strip with four selected variable OGs and one domain-variable OG. Variable-OG presence is evaluated in 44 non-edge-complete CC00231 BGCs or 50 non-edge-complete CC00158 BGCs.

In these matrices, purple denotes presence, white denotes absence in an evaluable complete BGC, and grey denotes absence of the CC or a non-evaluable BGC. The final columns map the Pfam architecture states of OG0004357 and OG0003621 across all genomes.

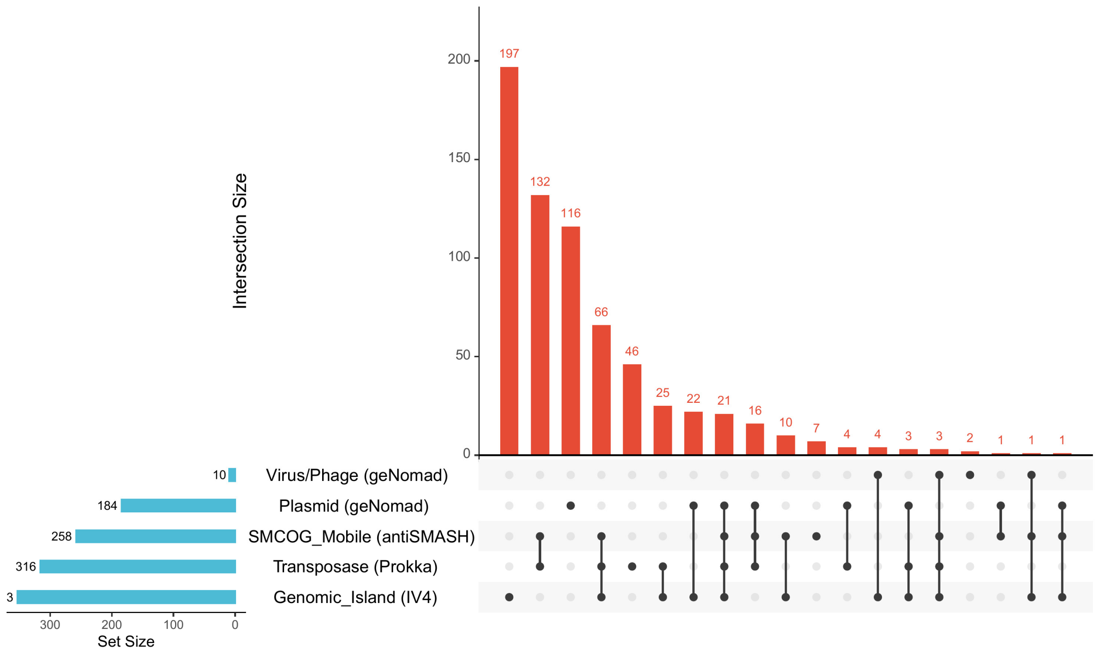

Figure S9. Intersection structure of five evidence dimensions used to classify MGE-associated BGCs.

The UpSet plot summarizes genomic-island overlap, nearby transposase or MGE genes, antiSMASH mobile-element annotations, plasmid-region overlap, and phage or viral-region overlap. Horizontal bars show set sizes and vertical bars show exact intersections.

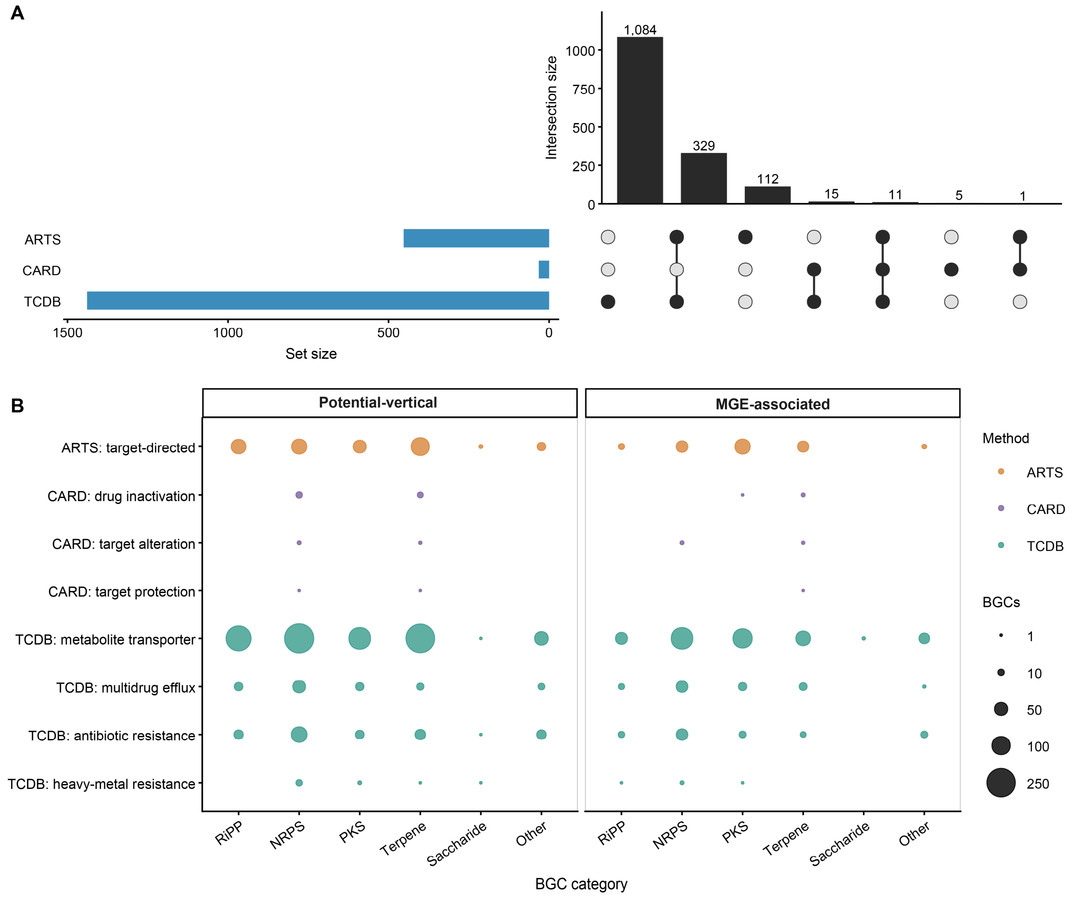

Figure S10. Intersection and class-level distribution of BGC-linked resistance evidence.

(A) UpSet plot of 1,557 BGCs linked to at least one prediction from ARTS, CARD, or TCDB within the BGC or its 10-kb flanks. ARTS, CARD, and TCDB linked to 453, 32, and 1,439 BGCs, respectively; bars show exact method intersections. (B) Numbers of distinct BGCs carrying each resistance- or transporter-associated feature across BGC categories, separated into potential-vertical and MGE-associated groups

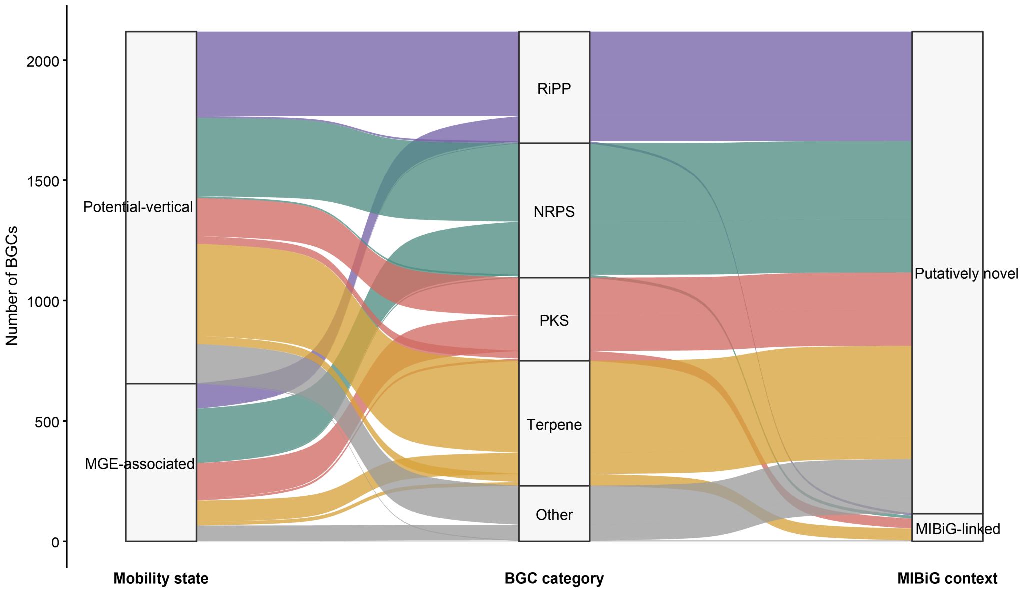

Figure S11. Mobility-associated flow of the *Lentzea* secondary metabolome.

The alluvial plot connects mobility state, BGC category, and MIBiG context for 2,120 *Lentzea* BGCs at BiG-SCAPE cutoff 0.20. Flow width represents the number of BGCs. The dataset includes 1,465 potential-vertical and 655 MGE-associated BGCs; the seven saccharide BGCs are grouped with Other for readability.

Supplementary materials files

{ TC "Data S1. Genome metadata, quality control and dataset membership." \f C \l "1" }Data S1. Genome metadata, quality control and dataset membership.

Table S2. Genome metadata, quality metrics and dataset membership.

{ TC "Data S2. Pangenome composition and functional analysis." \f C \l "1" }Data S2. Pangenome composition and functional analysis.

Table S3. Pangenome gene counts by compartment.

Table S4. COG composition across pangenome compartments.

Table S5. Core-versus-accessory COG enrichment statistics.

{ TC "Data S3. Family-scale BGC profiling, novelty and genus specificity." \f C \l "1" }Data S3. Family-scale BGC profiling, novelty and genus specificity.

Table S6. antiSMASH-predicted BGC regions.

Table S7. MIBiG similarity hits for predicted BGCs.

Table S8. Putatively novel BGC labels.

Table S9. Genome-level BGC abundance and assembly features.

Table S10. Genome size-BGC abundance correlations.

Table S11. Empirical BiG-SCAPE cutoff sensitivity metrics.

Table S12. Genus-specific BGC connected components and conservation tiers.

Table S13. Genus-level profiles of putatively novel BGCs.

{ TC "Data S4. Chromosome-stable candidate prioritization and microvariation." \f C \l "1" }Data S4. Potential-vertical candidate BGC conservation and domain architecture.

Table S14. Potential-vertical candidate selection and family-scale validation.

Table S15. Complete domain-variable-OG architecture assignments for CC00231 and CC00158.

Table S16. Recurrent domain-architecture events across potential-vertical candidates.

{ TC "Data S5. MGE-associated and resistance-linked BGC analyses." \f C \l "1" }Data S5. MGE-associated and resistance-linked BGC analyses.

Table S17. Five-dimensional MGE evidence for individual *Lentzea* BGCs.

Table S18. Statistical tests of MGE association across BGC strata.

Table S19. MGE classification robustness across BiG-SCAPE cutoffs.

Table S20. Resistance and transporter associations by BGC class.

References

1. Parks, D.H.; Imelfort, M.; Skennerton, C.T.; Hugenholtz, P.; Tyson, G.W. CheckM: assessing the quality of microbial genomes recovered from isolates, single cells, and metagenomes. *Genome Res.* **2015**, *25*, 1043-1055, doi: 10.1101/gr.186072.114.
2. Chaumeil, P.; Mussig, A.J.; Hugenholtz, P.; Parks, D.H. GTDB-Tk v2: memory friendly classification with the genome taxonomy database. *Bioinformatics* **2022**, *38*, 5315-5316, doi: 10.1093/bioinformatics/btac672.
3. Parks, D.H.; Chuvochina, M.; Rinke, C.; Mussig, A.J.; Chaumeil, P.; Hugenholtz, P. GTDB: an ongoing census of bacterial and archaeal diversity through a phylogenetically consistent, rank normalized and complete genome-based taxonomy. *Nucleic. Acids. Res.* **2022**, *50*, D785-D794, doi: 10.1093/nar/gkab776.
4. Olm, M.R.; Brown, C.T.; Brooks, B.; Banfield, J.F.; Lawrence Berkeley National Laboratory Lbnl, B.C.U.S. dRep: a tool for fast and accurate genomic comparisons that enables improved genome recovery from metagenomes through de-replication. *The Isme Journal* **2017**, *11*, 2864-2868, doi: 10.1038/ismej.2017.126.
5. Jain, C.; Rodriguez-R, L.M.; Phillippy, A.M.; Konstantinidis, K.T.; Aluru, S. High throughput ANI analysis of 90K prokaryotic genomes reveals clear species boundaries. *Nat. Commun.* **2018**, *9*, 5114, doi: 10.1038/s41467-018-07641-9.
6. Seemann, T. Prokka: rapid prokaryotic genome annotation. *Bioinformatics* **2014**, *30*, 2068-2069, doi: 10.1093/bioinformatics/btu153.
7. Cantalapiedra, C.P.; Hernández-Plaza, A.; Letunic, I.; Bork, P.; Huerta-Cepas, J. eggNOG-mapper v2: Functional Annotation, Orthology Assignments, and Domain Prediction at the Metagenomic Scale. *Mol. Biol. Evol.* **2021**, *38*, 5825-5829, doi: 10.1093/molbev/msab293.
8. Huerta-Cepas, J.; Szklarczyk, D.; Heller, D.; Hernández-Plaza, A.; Forslund, S.K.; Cook, H.; Mende, D.R.; Letunic, I.; Rattei, T.; Jensen, L.J.; et al. eggNOG 5.0: a hierarchical, functionally and phylogenetically annotated orthology resource based on 5090 organisms and 2502 viruses. *Nucleic. Acids. Res.* **2019**, *47*, D309-D314, doi: 10.1093/nar/gky1085.
9. Emms, D.M.; Kelly, S. OrthoFinder: phylogenetic orthology inference for comparative genomics. *Genome Biol.* **2019**, *20*, 238, doi: 10.1186/s13059-019-1832-y.
10. Buchfink, B.; Reuter, K.; Drost, H. Sensitive protein alignments at tree-of-life scale using DIAMOND. *Nat. Methods* **2021**, *18*, 366-368, doi: 10.1038/s41592-021-01101-x.
11. Katoh, K.; Standley, D.M. MAFFT multiple sequence alignment software version 7: improvements in performance and usability. *Mol. Biol. Evol.* **2013**, *30*, 772-780, doi: 10.1093/molbev/mst010.
12. Price, M.N.; Dehal, P.S.; Arkin, A.P. FastTree 2-Approximately Maximum-Likelihood Trees for Large Alignments. *Plos One* **2010**, *5*, 10, doi: 10.1371/journal.pone.0009490.
13. Lee, M.D. GToTree: a user-friendly workflow for phylogenomics. *Bioinformatics* **2019**, *35*, 4162-4164, doi: 10.1093/bioinformatics/btz188.
14. Wong, T.K.F.; Ly-Trong, N.; Ren, H.; Demotte, P.; Baños, H.; Roger, A.J.; Susko, E.; Bielow, C.; De Maio, N.; Goldman, N.; et al. IQ-TREE 3: Phylogenomic Inference Software using Complex Evolutionary Models. *Mol. Biol. Evol.* **2026**, msag117, doi: 10.1093/molbev/msag117.
15. Pritchard, L.; Glover, R.H.; Humphris, S.; Elphinstone, J.G.; Toth, I.K. Genomics and

taxonomy in diagnostics for food security: soft-rotting enterobacterial plant pathogens. *Anal. Methods* **2016**, *8*, 12-24, doi: 10.1039/C5AY02550H.

16. Blin, K.; Shaw, S.; Vader, L.; Szenei, J.; Reitz, Z.L.; Augustijn, H.E.; Cediel-Becerra, J.D.D.; de Crécy-Lagard, V.; Koetsier, R.A.; Williams, S.E.; et al. antiSMASH 8.0: extended gene cluster detection capabilities and analyses of chemistry, enzymology, and regulation. *Nucleic. Acids. Res.* **2025**, *53*, W32-W38, doi: 10.1093/nar/gkaf334.
17. Navarro-Muñoz, J.C.; Selem-Mojica, N.; Mullowney, M.W.; Kautsar, S.A.; Tryon, J.H.; Parkinson, E.I.; De Los Santos, E.L.C.; Yeong, M.; Cruz-Morales, P.; Abubucker, S.; et al. A computational framework to explore large-scale biosynthetic diversity. *Nat. Chem. Biol.* **2020**, *16*, 60-68, doi: 10.1038/s41589-019-0400-9.
18. Draisma, A.; Loureiro, C.; Louwen, N.L.L.; Kautsar, S.A.; Navarro-Muñoz, J.C.; Doering, D.T.; Mouncey, N.J.; Medema, M.H. BiG-SCAPE 2.0 and BiG-SLiCE 2.0: scalable, accurate and interactive sequence clustering of metabolic gene clusters. *Nat. Commun.* **2026**, *17*, 2000, doi: 10.1038/s41467-026-68733-5.
19. Zdouc, M.M.; Blin, K.; Louwen, N.L.L.; Navarro, J.; Loureiro, C.; Bader, C.D.; Bailey, C.B.; Barra, L.; Booth, T.J.; Bozhüyük, K.A.J.; et al. MIBiG 4.0: advancing biosynthetic gene cluster curation through global collaboration. *Nucleic. Acids. Res.* **2025**, *53*, D678-D690, doi: 10.1093/nar/gkae1115.
20. Gilchrist, C.L.M.; Chooi, Y. clinker & clustermap.js: automatic generation of gene cluster comparison figures. *Bioinformatics (Oxford, England)* **2021**, *37*, 2473-2475, doi: 10.1093/bioinformatics/btab007.
21. Blum, M.; Andreeva, A.; Florentino, L.C.; Chuguransky, S.R.; Grego, T.; Hobbs, E.; Pinto, B.L.; Orr, A.; Paysan-Lafosse, T.; Ponamareva, I.; et al. InterPro: the protein sequence classification resource in 2025. *Nucleic. Acids. Res.* **2025**, *53*, D444-D456, doi: 10.1093/nar/gkae1082.
22. Bertelli, C.; Laird, M.R.; Williams, K.P.; Lau, B.Y.; Hoad, G.; Winsor, G.L.; Brinkman, F.S. IslandViewer 4: expanded prediction of genomic islands for larger-scale datasets. *Nucleic. Acids. Res.* **2017**, *45*, W30-W35, doi: 10.1093/nar/gkx343.
23. Camargo, A.P.; Roux, S.; Schulz, F.; Babinski, M.; Xu, Y.; Hu, B.; Chain, P.S.G.; Nayfach, S.; Kyrpides, N.C. Identification of mobile genetic elements with geNomad. *Nat. Biotechnol.* **2024**, *42*, 1303-1312, doi: 10.1038/s41587-023-01953-y.
24. Mungan, M.D.; Alanjary, M.; Blin, K.; Weber, T.; Medema, M.H.; Ziemert, N. ARTS 2.0: feature updates and expansion of the Antibiotic Resistant Target Seeker for comparative genome mining. *Nucleic. Acids. Res.* **2020**, *48*, W546-W552, doi: 10.1093/nar/gkaa374.
25. Alcock, B.P.; Huynh, W.; Chalil, R.; Smith, K.W.; Raphenya, A.R.; Wlodarski, M.A.; Edalatmand, A.; Petkau, A.; Syed, S.A.; Tsang, K.K.; et al. CARD 2023: expanded curation, support for machine learning, and resistome prediction at the Comprehensive Antibiotic Resistance Database. *Nucleic. Acids. Res.* **2023**, *51*, D690-D699, doi: 10.1093/nar/gkac920.
26. Saier, M.H.; Reddy, V.S.; Moreno-Hagelsieb, G.; Hendargo, K.J.; Zhang, Y.; Iddamsetty, V.; Lam, K.J.K.; Tian, N.; Russum, S.; Wang, J.; et al. The Transporter Classification Database (TCDB): 2021 update. *Nucleic. Acids. Res.* **2021**, *49*, D461-D467, doi: 10.1093/nar/gkaa1004.
27. Liu, D.; Zhang, Y.; Fan, G.; Sun, D.; Zhang, X.; Yu, Z.; Wang, J.; Wu, L.; Shi, W.; Ma, J. IPGA: A handy integrated prokaryotes genome and pan-genome analysis web service. *Imeta* **2022**, *1*, e55, doi: 10.1002/imt2.55.

28. Shannon, P.; Markiel, A.; Ozier, O.; Baliga, N.S.; Wang, J.T.; Ramage, D.; Amin, N.; Schwikowski, B.; Ideker, T. Cytoscape: A Software Environment for Integrated Models of Biomolecular Interaction Networks; Genome Res., 2003; Volume 13, pp. 2498-2504.
